# Multi-strategy secretary bird optimization algorithm for UAV path planning in complex environment

**DOI:** 10.1038/s41598-025-28822-9

**Published:** 2025-11-29

**Authors:** Le Feng, Huanxi Liu, Zhifu He, Hanhao Ye, Sen Yang

**Affiliations:** 1 Huaneng Clean Energy Research Institute, Beijing, 100000 China; 2 Naval University of Engineering, Wuhan, 430030 China; 3 Liaoning Technical University, Huludao, 430030 China; 4 Fuzhou University, Fuzhou, 350108 China; 5 Southern University of Science and Technology, Shenzhen, 518055 China; 6 National Energy Technology Center for Hydropower Efficient Use and Dam Safety, Beijing, 100000 China; 7 National Energy R&D Center of Offshore Wind Power Engineering and Operation, Beijing, 100000 China

**Keywords:** Secretary bird optimization algorithm, Global exploration and local exploration, Pooling mechanism, Dynamic fitness distance balance strategy, UAV path planning, Engineering, Mathematics and computing

## Abstract

This paper proposes a UAV path planning method based on a Multi-strategy Secretary Bird Optimization Algorithm (MSBOA) to address the challenges of navigating complex terrain. First, a pooling mechanism is introduced to enhance population diversity and improve the algorithm’s optimization capabilities, balancing global exploration and local exploitation. Second, a dynamic fitness distance balance technique is incorporated to balance exploration and exploitation, preventing the population from becoming trapped in local optima while improving convergence accuracy. Finally, a greedy selection-based centroid reverse learning approach is used to update the population, enhancing the algorithm’s exploratory performance. To validate the effectiveness of the proposed improved algorithm, the proposed MSBOA is compared with classical and advanced intelligent algorithms by solving the CEC2017 benchmark test functions and a designed UAV environment model. Comparative analysis of simulation results indicates that the proposed MSBOA converges faster and achieves higher accuracy than the traditional SBOA. It effectively handles complex UAV path planning problems, enabling the design of faster, shorter and safer flight paths. This further demonstrates the excellent performance of the multi-strategy SBOA in UAV path planning, highlighting its broad application prospects.

## Introduction

Drones, due to their high flexibility, safety, robustness, and reliability, are widely used in disaster prevention and mitigation, traffic regulation, search and rescue, and other fields. Path planning refers to the process of determining the optimal or near-optimal route for a drone from its starting point to its destination ^1,2^. Ensuring mission execution and environmental safety is crucial. A path that is highly safe, feasible, computationally efficient, and cost-effective can significantly enhance the completion efficiency of drone missions. The planned path must meet specific criteria to ensure optimality ^3^. For instance, in applications such as aerial photography, surveying, and surface inspection, the path should be as short as possible to minimize time and fuel consumption ^4,5^. However, for other tasks such as dynamic target search or surveillance and rescue, the path needs to be formulated based on different criteria, potentially requiring the maximization of detection probability or the minimization of flight time. Additionally, the path planning must consider environmental safety and drone performance constraints to ensure collision avoidance and compliance with requirements related to flight time, altitude, and fuel consumption ^6–8^. Therefore, achieving safe path planning that enables collision-free and feasible movements remains a challenging task.

The core objective of drone path planning is to generate an optimal path from the starting point to the destination while satisfying various constraints, typically involving the comprehensive optimization of metrics such as path length, threat avoidance, energy consumption, and trajectory smoothness ^9,10^. In recent years, researchers have proposed a variety of path planning methods, which can be broadly categorized into traditional methods and intelligent optimization methods. Traditional methods, such as the A* algorithm ^11,12^, RRT^13,14^, and Dijkstra’s algorithm ^15–17^, involve constructing a cost graph for local search. While they can quickly identify feasible paths, they are prone to falling into local optima in high-dimensional complex environments and struggle to balance multi-objective optimization requirements. In contrast, intelligent optimization algorithms achieve global optimization by simulating biological group behaviors or physical processes. They demonstrate significant advantages in handling complex constraints and multi-objective problems, making them a current research focus ^18,19^.

It is well-known that optimizing the flight path of UAVs typically requires optimization algorithms. These optimization methods generally fall into deterministic mathematical programming methods and stochastic metaheuristic algorithms ^20^. However, deterministic mathematical programming methods often struggle with nonlinear spaces ^21,22^. Metaheuristic algorithms can solve complex problems that were previously considered difficult. They generate optimal or near-optimal solutions while reducing computational complexity, making them a good alternative when deterministic algorithms produce inefficient solutions. Path planning is a challenging problem for deterministic techniques, prompting the use of many heuristic-based techniques to address this new problem. Generally, metaheuristic algorithms can be categorized into four types ^23,24^: Evolutionary Algorithms (EA), Physics-based Algorithms (PhA), Human-based (HB) Algorithms, and Swarm Intelligence (SI) Algorithms. Evolutionary Algorithms typically generate better new populations through combinations and mutations among individuals from previous generations. Examples include Genetic Algorithms (GA) inspired by Darwin’s theory of evolution ^25^, Differential Evolution (DE) based on natural selection and reproduction processes ^26^, Genetic Programming (GP), and Evolution Strategies (ES) inspired by biological evolution processes ^27^. Physics-based methods use rules derived from various physical phenomena in nature to find solutions. Examples include Central Force Optimization ^28^, Simulated Annealing (SA) based on the annealing process in metallurgy ^29^, Galaxy-based Optimization (GSO) inspired by galaxy movements ^30^, and Gravitational Search Algorithm (GSA) based on Newton’s laws of gravity and motion ^31^. Human-based (HB) algorithms simulate natural human behaviors to solve optimization problems. Examples include Teaching-Learning-Based Optimization (TLBO) which models the influence of a teacher on learners’ outputs ^32^. Swarm Intelligence (SI) algorithms are inspired by the collective behavior of animals. Examples include Particle Swarm Optimization (PSO) based on the foraging behavior of birds and fish ^33^, Ant Colony Optimization (ACO) inspired by the social foraging behavior of ants ^34^, Grey Wolf Optimizer (GWO) based on the social hierarchy and hunting behavior of grey wolves ^35^, Whale Optimization Algorithm (WOA) inspired by the hunting strategies of humpback whales, which involve search for prey, encircling, and spiral updating ^36^, Black Widow Optimization (BWO) inspired by the unique reproductive behavior of black widow spiders ^37^, Red-Billed Blue Magpie Optimizer (RBMO) inspired by the behaviors of searching, chasing, attacking prey, and food storing of red-billed blue magpies ^38^, Quantum Aviator Navigation Algorithm (QANA) inspired by the extraordinary navigational precision of migratory birds ^39^, Golden Jackal Optimization (GJO) based on the cooperative hunting behavior of golden jackals ^40^.

However, stochastic metaheuristic algorithms also have inherent drawbacks. David H. Wolpert and William G. Macready ^41^ introduced the No Free Lunch (NFL) theorem in 1997, logically proving that no metaheuristic algorithm can best solve all optimization problems. In other words, while intelligent algorithms can achieve desired results in specific optimization problems, they may perform poorly in others. Consequently, researchers are attempting to integrate different intelligent algorithms into UAV path planning to find better solutions ^42–45^. For instance, Fu et al. ^38^ proposed the Red-Billed Blue Magpie Optimizer (RBMO), inspired by the behaviors of red-billed blue magpies such as searching, chasing, attacking prey, and food storing, for 2D and 3D UAV path planning problems. Huang et al. developed a PSO algorithm based on cylindrical vectors with wave and sigmoid functions for UAV path planning ^46^. Qu et al. combined the Simplified Grey Wolf Optimizer (SGWO) and Modified Symbiotic Organisms Search (MSOS) to propose a novel hybrid Grey Wolf Optimizer for UAV path planning ^47^. Manh et al. introduced a new algorithm based on Spherical Vector Particle Swarm Optimization (SPSO) to address UAV path planning in complex environments with multiple threats ^48^. Zhang et al. ^3^ proposed a new multi-objective evolutionary algorithm with a dual constraint handling mechanism for multi-path UAV planning. Li et al. ^49^ developed a Fermat Point-based Group Particle Swarm Optimization (FP-GPSO) algorithm for UAV path planning, while Yu et al. ^50^devised a hybrid GWO and Differential Evolution (HGWODE) algorithm to solve UAV path planning problems.

These efforts reflect the ongoing research to overcome the limitations of single metaheuristic approaches and enhance the effectiveness of UAV path planning through hybrid and innovative algorithmic integrations.

The Secretary Bird Optimization Algorithm (SBOA), recently proposed by Fu et al. ^51^, is a swarm intelligence algorithm inspired by the hunting and survival behaviors of secretary birds. It is characterized by its simple structure and fast convergence. However, in UAV path planning within complex environments, SBOA faces issues such as imbalance between exploration and exploitation, slow convergence speed, tendency to get trapped in local optima, and low convergence accuracy. To address these problems, this paper proposes a Multi-Strategy Secretary Bird Optimization Algorithm (MSBOA) based on pooling mechanisms, dynamic fitness distance balancing techniques, and centroid reverse learning. This algorithm is applied to UAV path planning in complex environments. The main contributions of this paper are as follows:

1. The successful integration of pooling mechanisms, dynamic fitness distance balancing techniques, Preferential selecting search strategy, and centroid reverse learning into SBOA, enhancing its overall performance.
2. Performance validation of MSBOA through comparison with four classical algorithms and four recent algorithms on CEC2017 benchmark test functions.
3. Successful application of MSBOA to UAV path planning in eight different scenarios generated from two real digital elevation model (DEM) maps with varying complexities.

The remainder of this paper is organized as follows: Section "UAV mathematical model" introduces the UAV mathematical model; Section "Secretary bird optimization algorithm" briefly explains the principles of the SBOA; Section "Multi-strategy secretary bird optimization algorithm" provides a detailed description of the four strategy improvements in the MSBOA; Section "Experimental results and analysis" presents the experiments on CEC2017 benchmark test functions and the UAV path planning simulations; Section "Summary and Prospect" concludes the study and discusses future work.

## UAV mathematical model

3. In complex terrains, a UAV follows the path planning results to navigate from the starting point to the target point. During its flight, the UAV may encounter various obstacles such as terrain features, fire threats, and radar scanning areas, in addition to constraints like fuel consumption, maximum climb rate, and maximum turning capability. To ensure the safe and coordinated flight of the UAV, the path planning algorithm must determine the optimal path that connects the start and end points ^48,52^.

Assuming the drone maintains a predetermined flight speed, the path planning problem is thereby simplified to a static piecewise planning problem. Considering the operational requirements of the drone, the cost function for drone path planning is defined by computing the cost of distance traveled, altitude, threats encountered, and smoothness. Mathematically, it can be represented as follows:

1 $$\begin{array}{c}{F}_{4}\left({X}_{i}\right)=\sum_{k=1}^{4} {b}_{k}{F}_{k}({X}_{i})\end{array}$$

Here, ${b}_{k}$ represents the weighting coefficients, with this paper adopting ${b}_{1}={b}_{2}=1,{b}_{3}=10,{b}_{4}=1$, ${F}_{1}\left({X}_{i}\right)$ through ${F}_{4}\left({X}_{i}\right)$ respectively denote the cost functions associated with path length, threat encountered, altitude, and smoothness. The decision variables are denoted by ${X}_{i}$,comprising a list of $l$ waypoints represented as ${P}_{ij}=({x}_{ij},{y}_{ij},{z}_{ij})$, where ${P}_{ij}$ belongs to the operational space $O$ of the drone.

### Path distance cost

In order to ensure efficient operation of the drone, path planning needs to be optimized according to specific criteria tailored to different application scenarios. Given our primary focus on aerial photography, mapping, and surface inspection, we opt to minimize path length as our objective. The cost function for path length, ${F}_{1}$ is determined by the Euclidean distance between adjacent nodes and is calculated using Equation (2).

2 $$\begin{array}{c}{F}_{1}\left({X}_{i}\right)=\sum_{j=1}^{l-1} \Vert \overrightarrow{{P}_{ij}{P}_{i,j+1}}\Vert .\end{array}$$

In this equation, ${P}_{ij}$ and ${P}_{i,j+1}$ represent adjacent nodes, and the sum of $n-1$ segments between $n$ nodes constitutes the cost of path length.

### Safety and feasibility costs

**Fig. 1:**
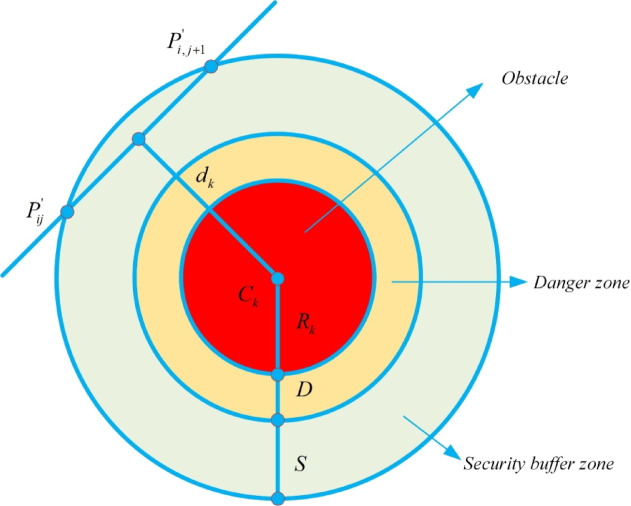
Safety constraint cost.

In addition to optimizing for minimum path length, path planning must also ensure the safe operation of the drone, which may encounter restricted zones known as threat areas (such as radar detection, anti-aircraft equipment attacks, or weather threats) ^53^. Considering the complexity of threat modeling and the difficulty in obtaining real data, this paper abstracts the threat environment by representing threat areas as cylindrical regions with a fixed radius. The radius of these cylindrical regions defines the effective threat range, and the purpose of defining threat areas is to ensure the drone’s operational safety by enabling it to successfully avoid these regions and complete its flight mission. Let $K$ denote the set of all threats, assuming each threat is contained within a cylindrical region with its projection center coordinates denoted by ${C}_{k}$ and radius ${R}_{k}$. Figure 1 illustrates the safety constraints. During drone flight, it is essential to avoid tall obstacles on the ground. The safety and feasibility cost of the path ${F}_{2}$ is calculated using Equations (3) and (4).

3 $$\begin{array}{c}{F}_{2}({X}_{i})=\sum_{j=1}^{l-1} \sum_{k=1}^{K} {T}_{k}(\overrightarrow{{P}_{ij}{P}_{i,j+1}})\end{array}$$

4 $$\begin{array}{c}{T}_{k}(\overrightarrow{{P}_{ij}{P}_{i,j+1}})=\left\{\begin{array}{cc}0,& \text{if }{d}_{k}>S+D+{R}_{k},\\ \left(S+D+{R}_{k}\right)-{d}_{k},& \text{if }D+{R}_{k}<{d}_{k}\le S+D+{R}_{k},\\ \infty ,& \text{if }{d}_{k}\le D+{R}_{k}.\end{array}\right.\end{array}$$

According to Equation (4) and as depicted in Fig. 1, when the distance ${d}_{k}$ is less than the sum of the danger zone radius $\left(D+{R}_{k}\right)$, the cost ${F}_{2}$ becomes infinite, indicating that the drone must maintain a safe distance from the danger zone during flight. Within the safety buffer zone, the cost increases as the drone gets closer to obstacles. When the distance ${d}_{k}$ exceeds $\left(S+D+{R}_{k}\right)$, the safety cost is zero, indicating the most ideal path for the drone.

### Flight altitude cost

**Fig. 2:**
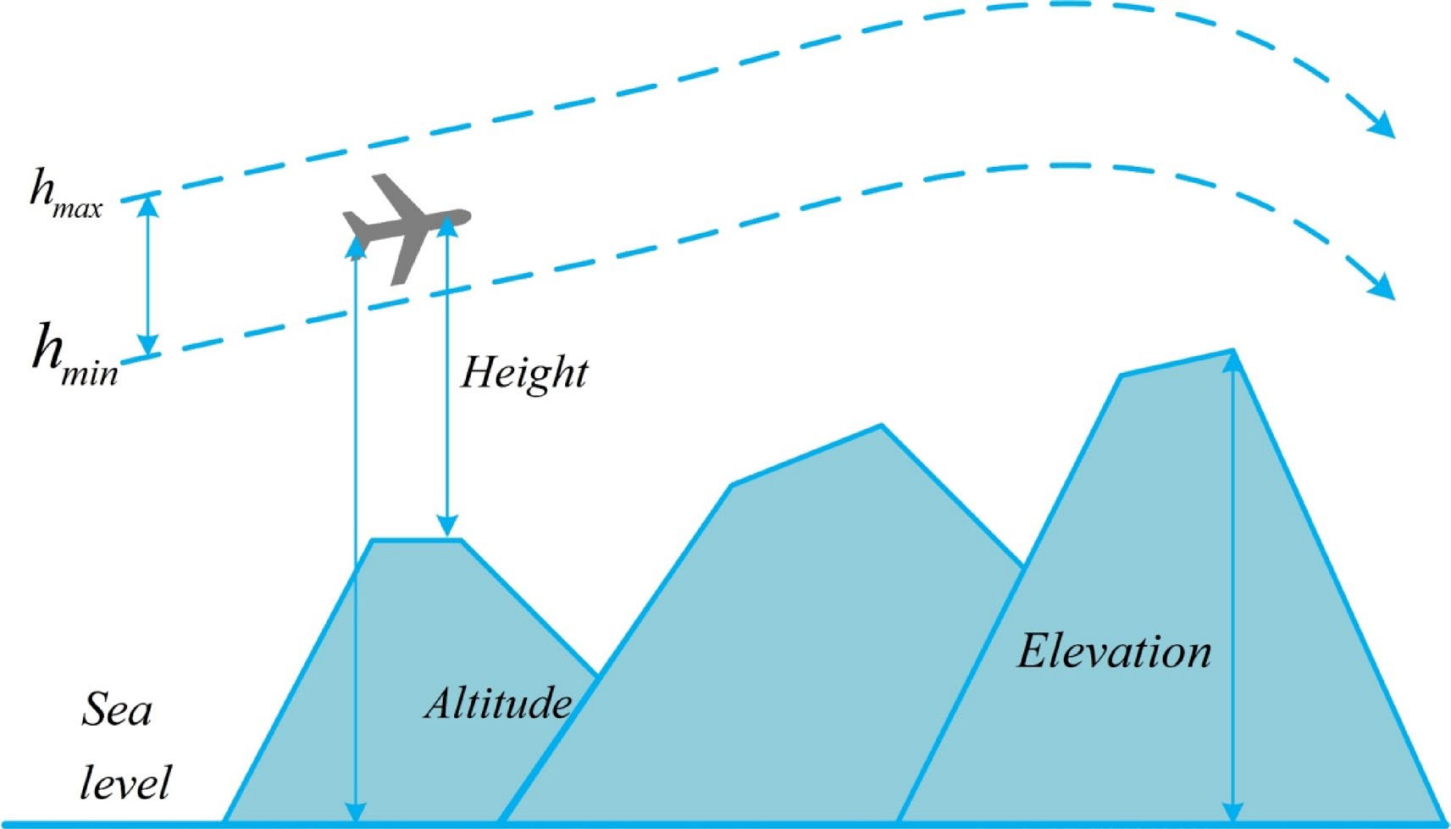
Highly constrained cost.

Subject to natural conditions and application-specific constraints, the altitude of drone flight is restricted. For instance, in tasks such as measurement and inspection, the altitude of flight is limited due to specific resolution and field of view requirements of cameras and inspection equipment ^54^. As illustrated in Fig. 2,the drone’s flight is constrained by a minimum altitude ${h}_{min}$ and a maximum altitude ${h}_{max}$. The cost associated with the altitude related to waypoint ${P}_{ij}$ is given by:

5 $$\begin{array}{c}{H}_{ij}=\left\{\begin{array}{c}|{h}_{ij}-\frac{\left({h}_{max}+{h}_{min}\right)}{2}|,{\text{i}}{\text{f}}{ h}_{min}\le {h}_{ij}\le {h}_{max},\\ \infty ,{\text{o}}{\text{t}}{\text{h}}{\text{e}}{\text{r}}{\text{w}}{\text{i}}{\text{s}}{\text{e}}.\end{array}\right.\end{array}$$

In Equation (5), ${h}_{ij}$ represents the flight altitude relative to the ground, as depicted in Fig. 2. It is evident that ${H}_{ij}$ maintains an average altitude and penalizes values that exceed the specified range. The summation of ${H}_{ij}$ for all waypoints yields the altitude cost ${F}_{3}$, as shown in Equation (6).

6 $$\begin{array}{c}{F}_{3}\left({X}_{i}\right)=\sum_{j=1}^{l} {H}_{ij}.\end{array}$$

### Path smoothing cost

**Fig. 3:**
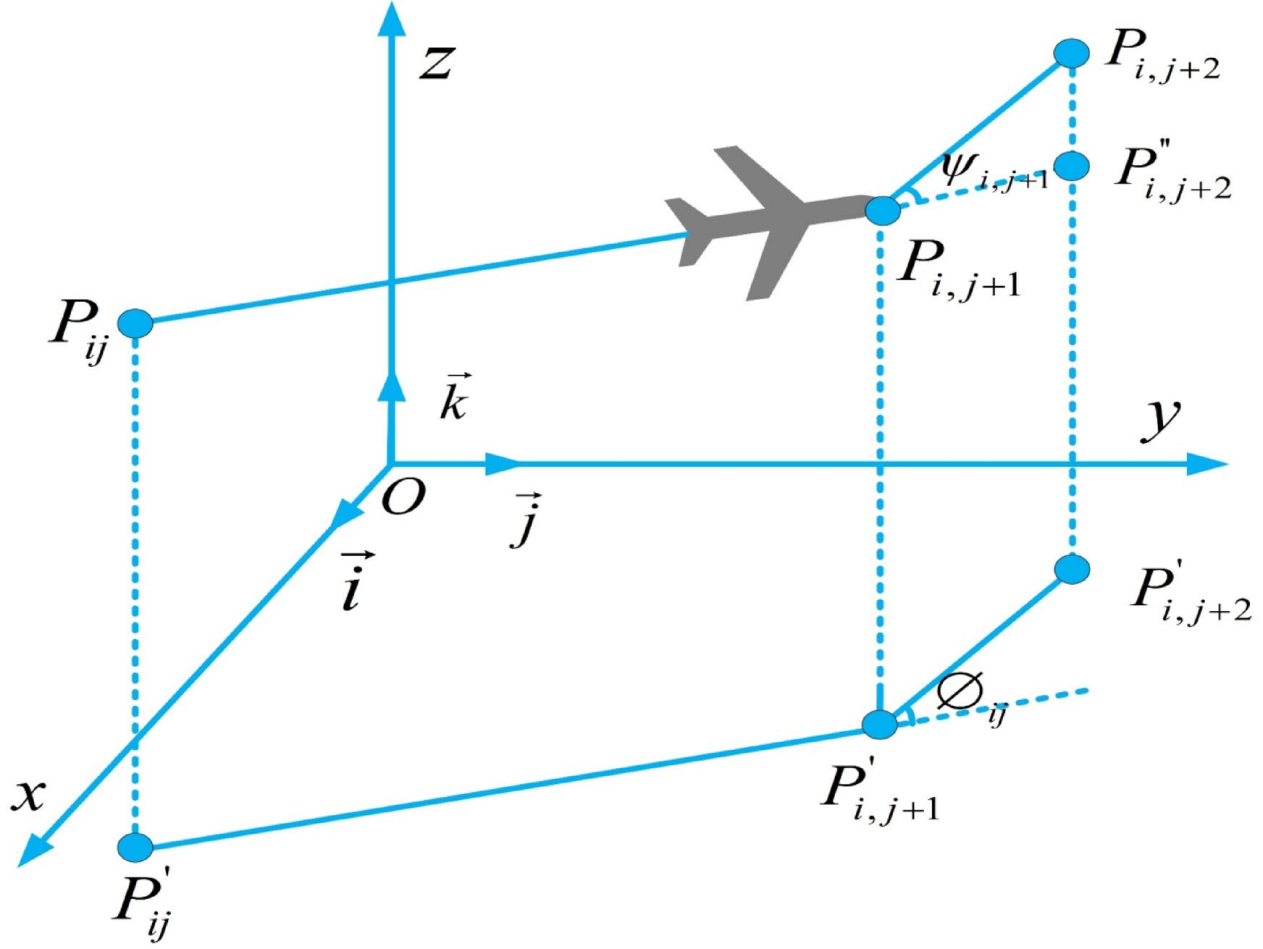
Turning Angle and climbing Angle constraints.

The primary control parameters for the drone’s flight angles include the horizontal heading angle and the vertical pitch angle. Both of these variable parameters must adhere to the actual angle constraints of the drone; otherwise, the trajectory planning model cannot generate feasible flight paths. As depicted in Fig. 3, the turning angle ${\phi }_{ij}$ is the angle in the horizontal plane ${O}_{xy}$ between two consecutive path segments $\overrightarrow {{P_{ij}{\prime} P_{i,j + 1}{\prime} }}$ and $\overrightarrow {{P_{i,j + 1}{\prime} P_{i,j + 2}{\prime} }}$.Let $\vec{k}$ be the unit vector in the z-axis direction, and the projection vector is calculated using Equation (7) ^54^.

7 $$\begin{array}{*{20}c} {\overrightarrow {{P_{ij}{\prime} P_{i,j + 1}{\prime} }} = \vec{k} \times \left( {\overrightarrow {{P_{ij} P_{i,j + 1} }} \times \vec{k}} \right).} \\ \end{array}$$

Therefore, the formula for the turning angle can be obtained as shown in Equation (8).

8 $$\begin{array}{*{20}c} {\phi_{ij} = \arctan \left( {\frac{{\overrightarrow {{P_{ij}{\prime} P_{i,j + 1}{\prime} }} \times \overrightarrow {{P_{i,j + 1}{\prime} P_{i,j + 2}{\prime} }} }}{{\overrightarrow {{P_{ij}{\prime} P_{i,j + 1}{\prime} }} .\overrightarrow {{P_{i,j + 1}{\prime} P_{i,j + 2}{\prime} }} }}} \right),0 < \phi_{ij} < 30^\circ .} \\ \end{array}$$

${\psi }_{ij}$ represents the angle between the projection segment $\left( {\overrightarrow {{P_{ij}{\prime} P_{i,j + 1}{\prime} }} } \right)$ and the path segment $\left( {\overrightarrow {{P_{ij}{\prime} P_{i,j + 1}{\prime} }} } \right)$ on the horizontal plane. It is calculated using Equation (9) ^55^.

9 $$\begin{array}{*{20}c} {\psi_{ij} = \arctan \left( {\frac{{z_{i,j + 1} - z_{ij} }}{{\overrightarrow {{P_{ij}{\prime} P_{i,j + 1}{\prime} }} }}} \right),0 < \psi_{ij} < 15^\circ .} \\ \end{array}$$

Finally, the smoothness cost ${F}_{4}$ is calculated using Equation (10).

10 $$\begin{array}{c}{F}_{4}\left({X}_{i}\right)={\varphi }_{1}\sum_{j=1}^{l-2} {\phi }_{ij}+{\varphi }_{2}\sum_{j=1}^{l-1} \mid {\psi }_{ij}-{\psi }_{i,j-1}\mid .\end{array}$$

where ${\varphi }_{1}$ and ${\varphi }_{2}$ are the penalty coefficients for the turning angle and the climbing angle, respectively, and are taken as 0.5 and 0.7, respectively^55^.

## Secretary bird optimization algorithm

### Initialization

The SBOA (Secretary Bird Optimization Algorithm) method belongs to the category of population-based metaheuristic methods, akin to other heuristic algorithms. Like other heuristic algorithms, the SBOA algorithm also randomly generates a set of candidate solutions within the search space.

11 $$\begin{array}{c}X={\left[\begin{array}{cc}\begin{array}{ccc}{x}_{\text{1,1}}& {x}_{\text{1,2}}& \cdots \\ {x}_{\text{2,1}}& {x}_{\text{2,2}}& \cdots \\ \vdots & \vdots & \ddots \end{array}& \begin{array}{ccc}{x}_{1,j}& \cdots & {x}_{1,Dim}\\ {x}_{2,j}& \cdots & {x}_{2,Dim}\\ \vdots & \ddots & \vdots \end{array}\\ \begin{array}{ccc}{x}_{i,1}& {x}_{i,2}& \cdots \\ \vdots & \vdots & \ddots \\ {x}_{N,1}& {x}_{N,2}& \cdots \end{array}& \begin{array}{ccc}{x}_{i,\text{j}}& \cdots & {x}_{i,Dim}\\ \vdots & \ddots & \vdots \\ {x}_{N,j}& \cdots & {x}_{N,Dim}\end{array}\end{array}\right]}_{N\times Dim }\end{array}$$

Where $X$ represents the population of secretary birds, ${X}_{i}$ denotes the position of the ${i}^{th}$ secretary bird, ${x}_{i,j}$ represents the positional information of the ${j}^{th}$ problem variable of the ${i}^{th}$ secretary bird, $N$ represents the population size, and $Dim$ represents the dimensionality of the problem variables.

The initial positional information of the secretary birds is randomly determined based on Equation (12):

12 $$\begin{array}{c}{x}_{i,j}=\left({upper}_{j}-{lower}_{j}\right)\times {r}_{1}+{lower}_{j}\end{array}$$

In this equation, ${x}_{i,j}$ represents the initial value of the ${j}^{th}$ decision variable for the ${i}^{th}$ candidate solution;

${upper}_{j}$ and ${lower}_{j}$ are the maximum and minimum bounds; ${r}_{1}$ is a random number in the range (0, 1).

### Hunting behavior (exploration)

The hunting behavior of secretary birds is typically divided into three stages: searching for prey $\left({P}_{1}\right)$, consuming prey $\left({P}_{2}\right)$, and attacking prey $\left({P}_{3}\right)$. During the searching for prey stage, the secretary bird seeks potential prey. Once the prey is identified, it enters the consuming prey stage, where it exhausts the prey’s stamina. With keen judgment of its actions towards the prey, the bird hovers, jumps, and provokes near the snake, gradually depleting the prey’s stamina until it launches an attack. This process is modeled using Equations (13) and (14).

13 $$\begin{array}{c}{x}_{i,j}^{new1}=\left\{\begin{array}{c}{P}_{1}:{ x}_{i,j}+{r}_{2}\times {(x}_{r\_1}-{x}_{{r}_{2}}), if t<\frac{1}{3}T\\ {P}_{2}:{x}_{best}+exp\left({\left(\frac{t}{T}\right)}^{4}\right)\times \left(RB-0.5\right)\times \left({x}_{best}-{x}_{i,j}\right), if \frac{1}{3}T<t<\frac{2}{3}T\\ {P}_{3}:{ x}_{best}+{\left(1-\frac{t}{T}\right)}^{\left(2\times \frac{t}{T}\right)}\times {x}_{i,j}\times RL, else\end{array}\right.\end{array}$$

14 $$\begin{array}{c}{X}_{i}=\left\{\begin{array}{c}{ X}_{i}^{new1}, if {F}_{i}^{new1}<{F}_{i}\\ {X}_{i } ,else\end{array}\right.\end{array}$$

Here, $t$ represents the current iteration number, $T$ represents the maximum iteration number, ${x}_{i,j}^{new1}$ represents the new state of the ${i}^{th}$ secretary bird in the first stage, ${x}_{r1}$ and ${x}_{r2}$ are randomly selected candidate solutions for the first stage iteration, ${r}_{2}$ represents $1\times Dim$ array randomly generated from the interval [0,1], ${x}_{i,j}^{new1}$ represents the positional information for its ${j}^{th}$ dimension, ${F}_{i}^{new1}$ represents its objective function fitness value. $RB$ represents a $1\times Dim$ array randomly generated from a standard normal distribution (with mean 0 and standard deviation 1), ${x}_{best}$ represents the best solution obtained so far. $RL$ represents the Levy flight function, calculated using Equation (15).

15 $$\begin{array}{c}\left\{\begin{array}{c}RL=0.5\times Levy\left(Dim\right)\\ Levy\left(Dim\right)=0.01\times \frac{u\times \sigma }{|v{|}^{\frac{1}{\eta }}}\\ \sigma ={\left(\frac{\Gamma \left(1+\eta \right)\times \mathit{sin}\left(\frac{\pi \times \eta }{2}\right)}{\Gamma \left(\frac{1+\eta }{2}\right)\times \eta \times 2\left(\frac{\eta -1}{2}\right)}\right)}^{\frac{1}{\eta }}\end{array}\right.\end{array}$$

In this equation, $\eta$ is a fixed constant equal to 1.5. $u$ and $v$ are random numbers within the interval [0, 1], $\Gamma$ represents the gamma function, and $\eta$ also has a value of 1.5.

### Escape strategy (exploitation)

Secretary birds may encounter attacks from predators or attempts to steal their food. They are highly intelligent and often employ evasion strategies to protect themselves or their food. These strategies primarily involve two approaches: fleeing by flying or running $\left({S}_{1}\right)$ and camouflage using the colors or structures in the environment $\left({S}_{2}\right)$, making it harder for predators to detect them. This process is modeled using Equations (16) and (17).

16 $$\begin{array}{c}{x}_{i,j}^{new2}=\left\{\begin{array}{c}{{S}_{1}: x}_{best}+\left(2\times RB-1\right)\times {\left(1-\frac{t}{T}\right)}^{2}\times {x}_{i,j} , if q<{r}_{3}\\ {S}_{2}:{ x}_{i,j}+{r}_{4}\times \left({x}_{rand}-l\times {x}_{i,j}\right) , else\end{array}\right.\end{array}$$

17 $$\begin{array}{c}{X}_{i}=\left\{\begin{array}{c}{ X}_{i}^{new2}, if {F}_{i}^{new2}<{F}_{i}\\ {X}_{i } ,else\end{array}\right.\end{array}$$

In these equations, $q=0.5$, ${r}_{3}$ and ${r}_{4}$ represent a $1\times Dim$ array randomly generated from a normal distribution, ${x}_{rand}$ represents a randomly selected candidate solution for the current iteration, and $l$ denotes a random integer, either 1 or 2.

**Fig. 4:**
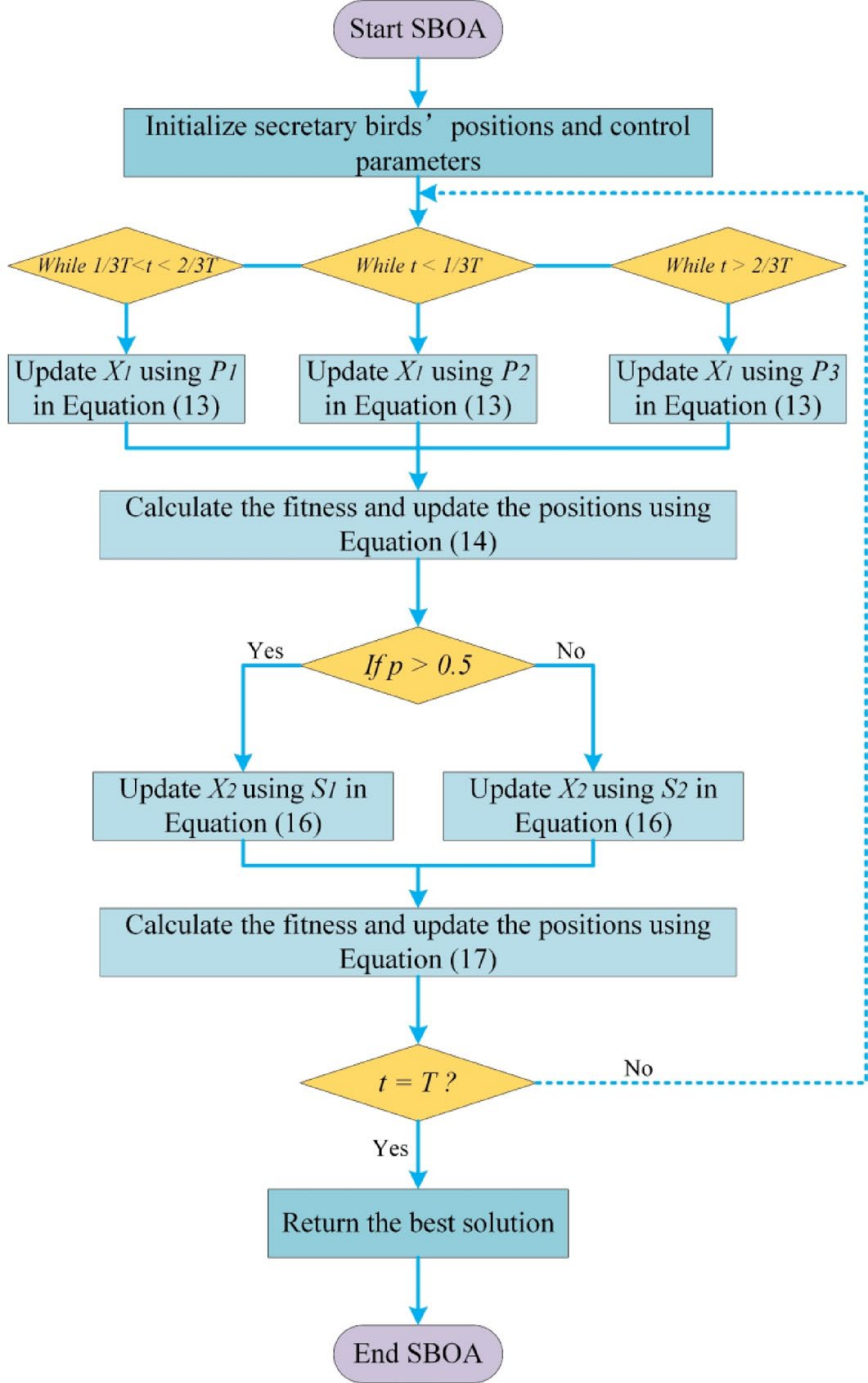
Flow chart of SBOA.

Based on the above description, the flowchart of the Secretary Bird Optimization Algorithm is illustrated in Fig. 4.

## Multi-strategy secretary bird optimization algorithm

To address the issues of poor local and global search capabilities, low population diversity, and consequently the inability to obtain high-quality solutions, this section proposes an improved version of the Secretary Bird Optimization Algorithm (SBOA), termed as the Multi-strategy Secretary Bird Optimization Algorithm (MSBOA). This enhancement is based on pooling mechanism, dynamic adaptability distance balancing technique, centroid reverse learning via greedy selection, and Preferential selecting search strategy.

### Pooling mechanism

Given a matrix $Pool=\left({P}_{1},{P}_{2},\dots ,{P}_{k}\right)$ of size $k$, where each member ${P}_{i}=\left({P}_{i,1},{P}_{i,2},\dots ,{P}_{i,Dim}\right)$ is generated at the end of each iteration using Equation (18), where ${X}_{brand}$ is computed using Equation (19) to generate a random position within the neighborhood of the best secretary bird ${X}_{best}$, and ${X}_{worst}$ is the worst solution obtained in the current iteration $t$. In this equation, ${B}_{i}$ is a binary random vector, ${\overline{B} }_{i}$ is its reverse vector such that the corresponding values of non-zero elements in ${B}_{i}$ are zero in ${\overline{B} }_{i}$ and vice versa. The pooling mechanism serves as a crossover operator, blending the worst solution with a promising one to generate a new solution, thus increasing diversity. Whenever the size of the $Pool$ is completed, the new solution will replace the current member of the $Pool$ ^56^.

18 $$\begin{array}{c}{P}_{i}={B}_{i}\times {X}_{brnd}+{\overline{B} }_{i}\times {X}_{worst}\end{array}$$

19 $$\begin{array}{c}{X}_{brand}={\xi }_{{best}_{min}}+{r}_{6}\times \left({\xi }_{{best}_{max}}-{\xi }_{{best}_{min}}\right)\end{array}$$

Where ${\xi }_{{best}_{min}}$ and ${\xi }_{{best}_{max}}$ are the lower and upper bounds of ${X}_{best}$, and ${r}_{6}$ is a uniformly distributed random number between 0 and 1.

Furthermore, in the evasion strategy camouflage $\left({S}_{1}\right)$ stage of the secretary bird, two individuals are randomly selected from the $Pool$ for mutation, enhancing population diversity and improving the algorithm’s optimization capability. The new strategy is represented as follows:

20 $$\begin{array}{c}{{S}_{1}: x}_{best}+\left(2\times RB-1\right)\times {\left(1-\frac{t}{T}\right)}^{2}\times \left(\left({{r}_{6}\times P}_{ran{d}_{1}}+{P}_{ran{d}_{2}}\right)\times 0.5\right)\end{array}$$

In this equation, ${P}_{ran{d}_{1}}$ and ${P}_{ran{d}_{2}}$ are individuals randomly selected from the Pool matrix in iteration $t$, and ${r}_{6}=2\times rand$ is a random number in the range [0,2].

### Dynamic fitness distance balance strategy

The Dynamic Fitness Distance Balance (DFDB) strategy is an improved version of the Fitness Distance Balance (FDB) method. FDB is a selection method aimed at effectively guiding the exploration and exploitation processes of the MHS algorithm during the search process ^57^. FDB is also a greedy method, where solution candidates are selected from the population based on their scores. What sets FDB apart from other selection methods is that it calculates scores for candidate solutions and selects based on these scores. In computing the scores, both the fitness value of the candidate solution and its distance from the best solution in the population are considered, ensuring that candidates with high fitness values are chosen ^58^. On the other hand, this also prevents the selection of a candidate solution very close to the best solution in the population, avoiding local optima. The implementation steps of the DFDB selection method are as follows:

a) When computing the score for each candidate solution in the population, both their fitness value and distance value are considered. Let ${X}_{i}=\left({x}_{i,1},{x}_{i,2},\dots ,{x}_{i,n-1},{x}_{i,n}\right)$ be the ${i}^{th}$ candidate solution in the population, and let ${X}_{best}=\left({x}_{1,best},{x}_{2,best},\dots ,{x}_{n-1,best},{x}_{n,best}\right)$ be the best solution in the population, where $\left(i=\text{1,2},\dots ,N\right)$. The Euclidean distance ${D}_{{X}_{i}}$ between the ${i}^{th}$ candidate solution and the best solution ${X}_{best}$ in the population is calculated as follows:

21 $$\begin{array}{c}{}_{i=1}{}^{n}\forall {X}_{i},{D}_{{X}_{i}}=\sqrt{{\left({x}_{i,1}-{X}_{best,1}\right)}^{2}+{\left({x}_{i,2}-{X}_{best,2}\right)}^{2}+\dots +{\left({x}_{i,N}-{X}_{best,n}\right)}^{2}}\end{array}$$

b) The distances of candidate solutions in the population can be represented by a vector ${D}_{X}$ as follows:

22 $$\begin{array}{c}{D}_{X}={\left[ \begin{array}{l}{d}_{1}\\ \vdots \\ {d}_{j}\\ \vdots \\ {d}_{m}\end{array}\right]}_{m\times 1}\end{array}$$

c) The fitness values and distance values of candidate solutions are normalized for calculating the score of each candidate solution. The formula for calculating the score is as follows:

23 $$\begin{array}{c}{\forall }_{i=1}^{N}{ X}_{i}, { S}_{{X}_{i}}=w\times norm{F}_{{X}_{i}}+(1-w)\times norm{D}_{{X}_{i}}\end{array}$$

In these equations, $w$ is the weighting coefficient determining the influence of fitness and distance values ($norm{F}_{{X}_{i}}$ and $norm{D}_{{X}_{i}}$) on the score calculation. In the DFDB method, $w=0.6\left(1-\frac{mod\left(t,round\left(T/10\right)\right)}{round\left(T/10\right)}\right)$. The DFDB score of solution candidates in the population is represented by the ${S}_{X}$ vector given by Equation (24).

24 $$\begin{array}{c}{S}_{X}={\left[ \begin{array}{l}{S}_{1}\\ \vdots \\ {S}_{j}\\ \vdots \\ {S}_{m}\end{array}\right]}_{m\times 1}\end{array}$$

To better balance the exploration and exploitation of the SBOA algorithm and address the issue of premature convergence, this paper integrates the DFDB technique into the SBOA algorithm. By dynamically adjusting the weights of fitness and distance between individuals, this integration aims to increase the diversity of the search, avoid premature convergence, and accelerate convergence speed. Consequently, this helps the algorithm explore the search space more effectively and find high-quality solutions.

### Centroid opposition-based learning with greedy selection

Greedy selection is a common optimization algorithm strategy, often used to select local optimal solutions to construct global optimal solutions. In algorithms like K-means clustering, greedy selection is used to choose initial centroids. However, in some cases, this greedy selection may lead to getting stuck in local optima or selecting suboptimal initial centroids. Centroid reverse learning, on the other hand, improves optimization efficiency by introducing reverse solutions of the best solution as feasible solutions, thereby expanding the search space. However, pure reverse learning may pose the risk of falling into local optima. We propose a strategy based on greedy selection of centroid reverse learning. The implementation steps are as follows:

After each iteration, generate a random integer $K$ from the interval $\left[1,N\right]$, where $N$ is the population size. Select the top $K$ individuals with the best fitness in the current population, denoted as $X=\left({X}_{1},{X}_{2}\dots {X}_{K-1},{X}_{K}\right)$, and compute the centroid of these $K$ individuals as follows:

25 $$\begin{array}{c}Barycenter=\frac{\sum_{i=1}^{K}{K}_{i}}{K},K=\text{1,2},\dots ,N\end{array}$$

Generate the reverse solutions for each individual at the centroid position as follows:

26 $$\begin{array}{c}{X}_{i}{\prime}=2\times Barycenter-{X}_{i},i=\text{1,2},\dots ,N-1,N\end{array}$$

Finally, use greedy selection to choose the top $N$ individuals with the best fitness values from the merged population $X\cup {X}{\prime}$ as the new population. Here, $X$ represents the initial population, and ${X}{\prime}$ represents the reverse solutions of $X$.

To address the slow convergence speed and low precision of the SBOA, this paper integrates a random centroid reverse learning mechanism into the SBOA algorithm for updating the population. This integration aims to enhance the quality and diversity of the population, ensuring that the algorithm can comprehensively optimize the search space and thus increase the probability of finding the global optimum.

### Preferential selecting search strategy

The Preferential Selecting Search (PSS) strategy enhances the exploration capability of the Secretary Bird Optimization Algorithm (SBOA) in searching for prey. This strategy is defined by Equation (27), where ${X}_{i}$ represents the current position of the ${i}^{th}$ secretary bird, ${P}_{ran{d}_{3}}$ and ${P}_{ran{d}_{4}}$ are individuals randomly selected from the Pool matrix in iteration $t$, and $Cauchy(i)$ is sampled from a Cauchy distribution computed by Equation (28). Because the Preferential Selecting Search strategy aims to enhance the exploration capability of SBOA, it requires a larger step size. By distributing the secretary birds in different regions of the search space, diverse solutions can be discovered. Therefore, the Cauchy distribution is employed, which has the characteristic of having long and flat tails, resulting in a high probability of generating large values. This distribution is utilized for the hunting attack stage of the secretary bird to enhance exploration ^59^. In the Chinese context, the utilization of the Cauchy distribution’s heavy-tailed property significantly improves search efficiency by increasing the probability of exploring distant regions in the solution space, thereby effectively preventing premature convergence and enhancing the algorithm’s global optimization capability.

27 $$\begin{array}{c}{x}_{i,j}^{new1}={x}_{best}+Cauchy\left(i\right)\times \left({P}_{ran{d}_{3}}-{P}_{ran{d}_{4}}\right), while t>\frac{2}{3}T\end{array}$$

28 $$\begin{array}{c}Cauchy=0.5+0.1\times tan\left(\pi \times rand\left(N,1\right)-0.5\right)\end{array}$$

In summary, the MSBOA updates the position information during the hunting and evasion stages using Equation (29) and Equation (30) respectively.

29 $$\begin{array}{c}{x}_{i,j}^{new1}=\left\{\begin{array}{c}{P}_{1}:{ x}_{i,j}+{r}_{2}\times {(x}_{r1}-{x}_{r2}), if t<\frac{1}{3}T\\ {P}_{2}:{x}_{best}+exp\left({\left(\frac{t}{T}\right)}^{4}\right)\times \left(RB-0.5\right)\times \left({x}_{best}-{x}_{i,j}\right), if \frac{1}{3}T<t<\frac{2}{3}T\\ {P}_{3}:{x}_{best}+Cauchy\left(i\right)\times \left({P}_{ran{d}_{3}}-{P}_{ran{d}_{4}}\right), else\end{array}\right.\end{array}$$

30 $$\begin{array}{c}{x}_{i,j}^{new2}=\left\{\begin{array}{c}{{S}_{1}: x}_{best}+\left(2\times RB-1\right)\times {\left(1-\frac{t}{T}\right)}^{2}\times \left(\left({{r}_{6}\times P}_{ran{d}_{1}}+{P}_{ran{d}_{2}}\right)\times 0.5 \right) , if q<{r}_{3}\\ {S}_{2}:{ x}_{i,j}+{r}_{4}\times \left({x}_{rand}-l\times {x}_{i,j}\right) , else\end{array}\right.\end{array}$$

The pseudocode for MSBOA is depicted as Algorithm 1.

**Algorithm 1:**
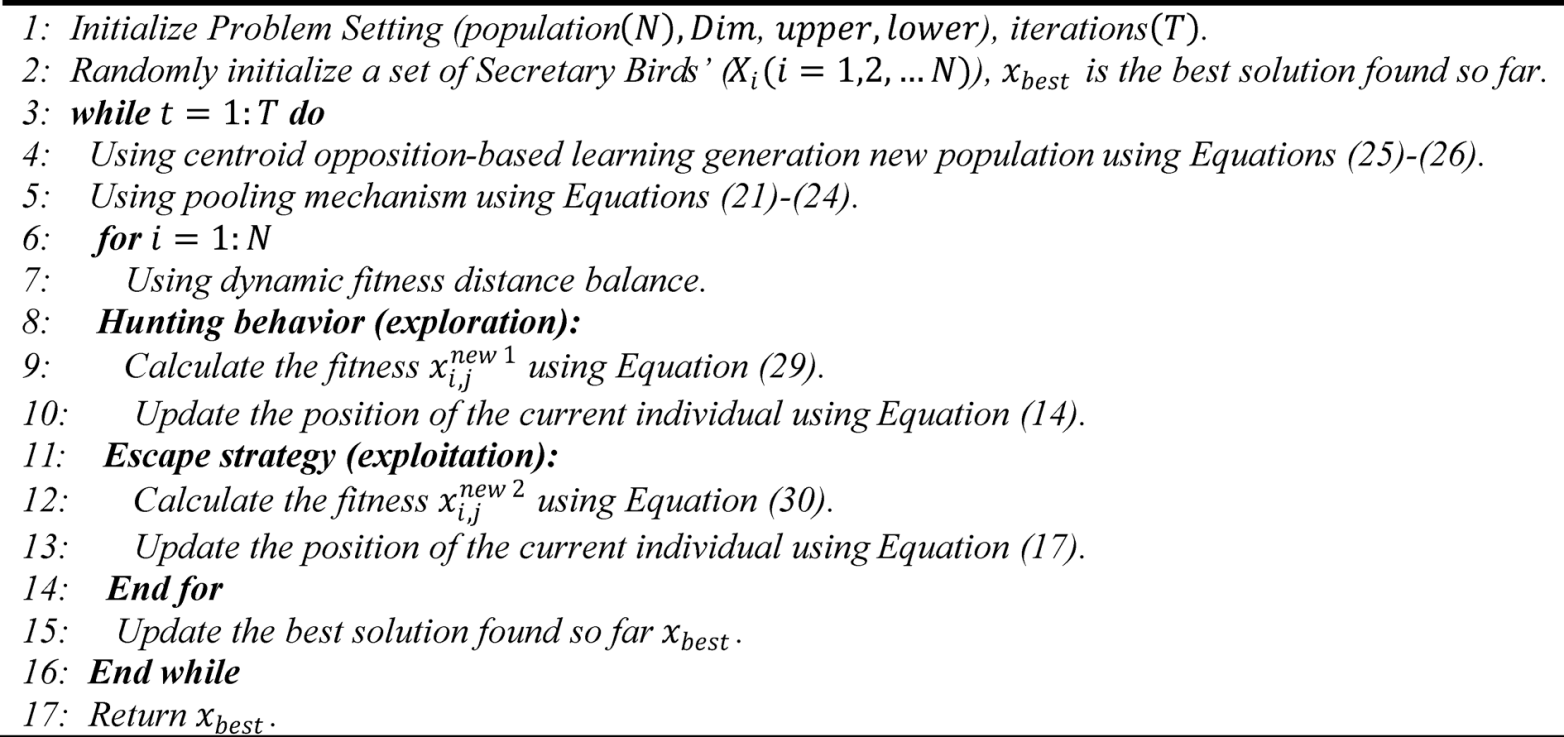
Pseudo-Code of MSBOA

### Computational time complexity of MSBOA

The performance of an algorithm is crucial, but it is equally important to evaluate its time complexity. In many optimization tasks, an algorithm must not only demonstrate excellent performance but also exhibit high real-time efficiency. Time complexity reflects how the algorithm’s runtime scales with the size of the input. Analyzing the time complexity of an optimization algorithm helps estimate its computational cost when handling large-scale problems. In the standard SBOA, the computational complexity of the defined control parameters is $O(N\times D)$, where $N$ represents the population size and $D$ denotes the problem dimension. During the initialization phase, the algorithm requires $O(N\times D)$ time. Furthermore, over $T$ iterations, the computational complexity for updating individual positions is $O(T\times N\times D)$.Therefore, the overall computational complexity of the SBOA algorithm can be expressed as $O(T\times N\times D)$. In the proposed MSBOA, we introduce two enhancement strategies: a pooling mechanism and centroid backpropagation learning. While these strategies do not change the asymptotic complexity order, they introduce additional computational overhead that affects actual runtime. Pooling Mechanism: Maintaining $k$ individuals and performing crossover operations adds an overhead of $O(k\times D)$ per iteration. Centroid Backpropagation Learning: Computing the centroid and backpropagated solutions for $K$ individuals introduces an additional cost of $O(K\times D)$ per iteration. Therefore, the overall computational complexity of MSBOA can be expressed as $O(T\times \left(N+k+K\right)\times D)$.

## Experimental results and analysis

In this chapter, to validate the effectiveness of MSBOA, its performance is evaluated using the CEC2017 benchmark tests ^60^ and the unmanned aerial vehicle (UAV) path planning problem described in Section "UAV mathematical model", and compared with eight other advanced comparative algorithms. The CEC2017 benchmark functions are presented in Table 1, classified into four types: unimodal, multimodal, hybrid, and composite functions. Unimodal functions are utilized to assess accuracy and convergence speed on simple optimization problems; multimodal functions evaluate the algorithm’s local exploration capability; and hybrid and composite functions assess the algorithm’s capability to handle complex continuous problems. The experiments were conducted on a computing environment with a Windows 11 operating system, hardware configuration consisting of a 13th Intel(R) Core (TM) i5-13400 2.5GHz processor, 16GB RAM, and MATLAB 2023b software tools.

**Table 1:** CEC2017 benchmark functions.

Type	ID	Function name	Rang	Dimension	${f}_{min}$
Unimodal	F1	Shifted and rotated bent cigar function	[−100,100]	30	100
	F2	Shifted and rotated sum of different power function	[−100,100]	30	200
	F3	Shifted and rotated zakharov function	[−100,100]	30	300
Multimodal	F4	Shifted and rotated rosenbrock’s function	[−100,100]	30	400
	F5	Shifted and rotated rastrigin’s function	[−100,100]	30	500
	F6	Shifted and rotated expanded scaffer’s F6 function	[−100,100]	30	600
	F7	Shifted and rotated lunacek Bi_Rastrigin function	[−100,100]	30	700
	F8	Shifted and rotated non-continuous rastrigin’s function	[−100,100]	30	800
	F9	Shifted and rotated levy function	[−100,100]	30	900
	F10	Shifted and rotated schwefel’s function	[−100,100]	30	1000
Hybrid	F11	Hybrid function 1 (N=3)	[−100,100]	30	1100
	F12	Hybrid function 2 (N=3)	[−100,100]	30	1200
	F13	Hybrid function 3 (N=3)	[−100,100]	30	1300
	F14	Hybrid function 4 (N=4)	[−100,100]	30	1400
	F15	Hybrid function 5 (N=4)	[−100,100]	30	1500
	F16	Hybrid function 6 (N=4)	[−100,100]	30	1600
	F17	Hybrid function 6 (N=5)	[−100,100]	30	1700
	F18	Hybrid function 6 (N=5)	[−100,100]	30	1800
	F19	Hybrid function 6 (N=5)	[−100,100]	30	1900
	F20	Hybrid function 6 (N=6)	[−100,100]	30	2000
Composition	F21	Composition function 1 (N=3)	[−100,100]	30	2100
	F22	Composition function 2 (N=3)	[−100,100]	30	2200
	F23	Composition function 3 (N=4)	[−100,100]	30	2300
	F24	Composition function 4 (N=4)	[−100,100]	30	2400
	F25	Composition function 5 (N=5)	[−100,100]	30	2500
	F26	Composition function 6 (N=5)	[−100,100]	30	2600
	F27	Composition function 7 (N=6)	[−100,100]	30	2700
	F28	Composition function 8 (N=6)	[−100,100]	30	2800
	F29	Composition function 9 (N=3)	[−100,100]	30	2900
	F30	Composition function 10 (N=3)	[−100,100]	30	3000

### Experimental results in CEC2017

**Fig. 5:**
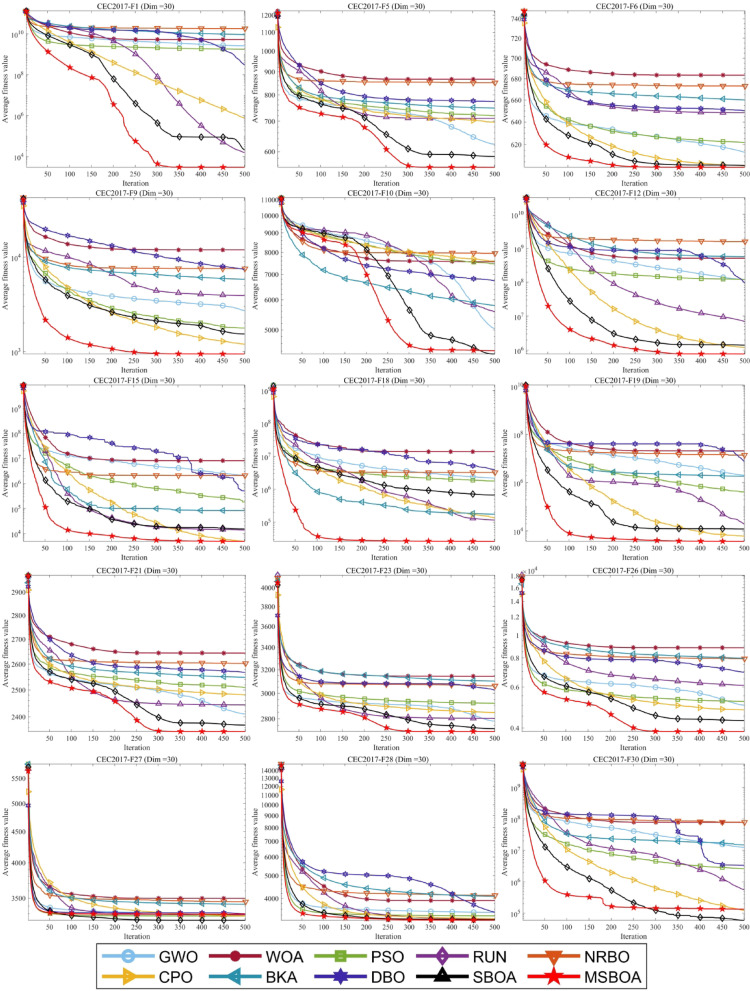
Different algorithms in CEC2017 benchmark test the convergence curve of the function.

**Fig. 6:**
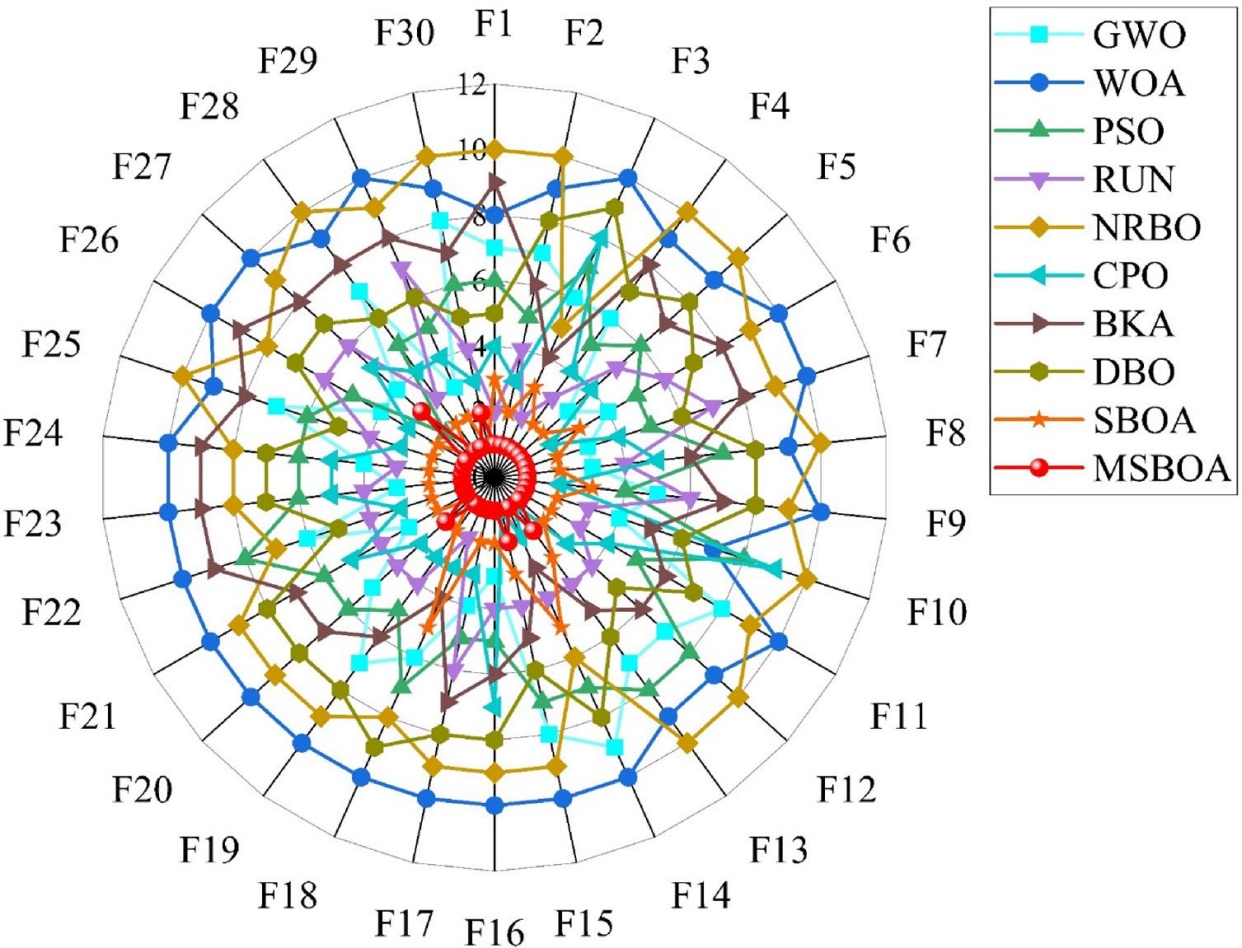
Ranking distribution of different algorithms.

In this section, MSBOA is compared with other advanced algorithms on the CEC2017 benchmark functions. All these functions are designed to minimize the objective function. The comparative algorithms include the classical algorithms Grey Wolf Optimizer (GWO) ^35^, Whale Optimization Algorithm (WOA) ^36^, Particle Swarm Optimization (PSO) ^33^, Runge Kutta optimizer (RUN) ^61^, as well as recently proposed advanced algorithms: Newton-Raphson-based optimizer (NRBO) ^62^, Crested Porcupine Optimizer (CPO) ^63^, Black-winged Kite Algorithm (BKA) ^64^, Dung Beetle Optimizer (DBO) ^65^, Secretary Bird Optimization Algorithm(SBOA) ^51^. The parameter settings for each algorithm are provided in Table 2. In the experiments, the population size is set to 30, and the maximum number of iterations is set to 500. To mitigate the influence of randomness and errors, each algorithm is independently run 50 times, calculating the mean and variance to obtain the final comparison results, with the best results highlighted in bold. Partial convergence curves of different algorithms on CEC2017 test functions are depicted in Fig. 5 and experimental statistical results are presented in Table 3. To visualize the ranking of each algorithm on each test function, radar charts as shown in Fig. 6 are plotted.

**Table 2:** Compare algorithm parameter settings.

Algorithms	Name of the parameter	Value of the parameter
GWO	$a$	Decrease linearly from 2 to 0
WOA	$r, l, a$	[0,1], [−1,1], [0,2]
PSO	${c}_{1},{c}_{2}, w$	1.49445, 1.49445, 0.9
RUN	$a, b$	20, 12
NRBO	$DF$	0.6
CPO	$\alpha , {N}_{min}, {T}_{f}, T$	0.1, 80, 0.5, 2
BKA	$P, r$	0.9, [0,1]
DBO	${P}_{percent}$	0.2
SBOA	$CF,K, {R}_{1},{R}_{2}$	$\left[\text{0,1}\right],\left\{1, 2\right\},[\text{0,1}],[\text{0,1}]$
MSBOA	${r}_{2},{r}_{4},{r}_{6},l$	$\left[\text{0,1}\right],[\text{0,1}],[\text{0,2}],\left\{1, 2\right\}$

**Table 3:** CEC2017 benchmark function experiment results.

ID	Metric	GWO	WOA	PSO	RUN	NRBO	CPO	BKA	DBO	SBOA	MSBOA
F1	Ave	2.5521E+09	5.1676E+09	1.2975E+09	1.5829E+04	1.7239E+10	7.4575E+05	9.4710E+09	2.3113E+08	2.1879E+04	**3.2249E+03**
	Std	1.5917E+09	1.9530E+09	9.1378E+08	9.1266E+03	4.3342E+09	9.8014E+05	7.0229E+09	1.1151E+08	1.8097E+04	**3.8036E+03**
F2	Ave	4.0289E+32	8.2322E+36	2.9751E+30	2.7176E+23	4.6834E+36	1.8199E+20	1.0178E+42	8.6724E+32	7.7868E+17	**3.2204E+13**
	Std	1.7244E+33	2.8943E+37	9.9001E+30	1.3680E+24	2.9952E+37	7.7437E+20	7.1968E+42	3.6762E+33	2.9641E+18	**1.8633E+14**
F3	Ave	6.0144E+04	2.6205E+05	6.5042E+04	1.0157E+04	5.5109E+04	6.2002E+04	3.6399E+04	9.6143E+04	2.6280E+04	**7.1860E+02**
	Std	1.2537E+04	5.3564E+04	1.8494E+04	4.4569E+03	8.8257E+03	1.1271E+04	1.7574E+04	3.3737E+04	7.7922E+03	**4.0956E+02**
F4	Ave	6.7258E+02	1.4485E+03	6.2187E+02	5.1226E+02	2.0695E+03	5.1834E+02	1.9701E+03	6.7182E+02	5.0641E+02	**5.0116E+02**
	Std	2.7615E+02	4.0016E+02	1.3550E+02	1.7083E+01	1.2754E+03	**1.6472E+01**	3.1434E+03	1.4519E+02	2.1733E+01	2.4724E+01
F5	Ave	6.2767E+02	8.6692E+02	7.1075E+02	6.9742E+02	8.4664E+02	6.9584E+02	7.4848E+02	7.5874E+02	5.8069E+02	**5.4985E+02**
	Std	4.4472E+01	6.0095E+01	2.4072E+01	4.4229E+01	3.9615E+01	**1.4194E+01**	5.4827E+01	5.1778E+01	1.8737E+01	1.5205E+01
F6	Ave	6.1261E+02	6.7918E+02	6.2024E+02	6.4705E+02	6.7147E+02	6.0204E+02	6.6045E+02	6.4863E+02	6.0302E+02	**6.0078E+02**
	Std	4.9031E+00	1.3122E+01	5.4580E+00	7.7242E+00	9.3481E+00	**9.1006E-01**	9.3761E+00	1.1820E+01	2.7897E+00	1.3956E+00
F7	Ave	9.0277E+02	1.3312E+03	9.9089E+02	1.0727E+03	1.2409E+03	9.4004E+02	1.2163E+03	1.0129E+03	8.6565E+02	**7.8644E+02**
	Std	5.3314E+01	7.6564E+01	2.5870E+01	8.0583E+01	7.8285E+01	**1.7529E+01**	7.0389E+01	8.1986E+01	4.4417E+01	1.9022E+01
F8	Ave	9.1281E+02	1.0832E+03	1.0052E+03	9.5476E+02	1.0847E+03	9.8175E+02	9.9398E+02	1.0325E+03	8.7531E+02	**8.4903E+02**
	Std	3.2507E+01	5.4220E+01	2.9185E+01	2.2757E+01	2.7402E+01	1.6924E+01	4.2688E+01	4.9446E+01	1.9276E+01	**1.6828E+01**
F9	Ave	2.6906E+03	1.0703E+04	2.3003E+03	3.8436E+03	7.7147E+03	1.2658E+03	5.2565E+03	6.3705E+03	1.5808E+03	**9.3402E+02**
	Std	1.2436E+03	3.1845E+03	1.4066E+03	7.4051E+02	1.4640E+03	3.1024E+02	1.1387E+03	2.1973E+03	5.5370E+02	**3.4029E+01**
F10	Ave	5.2700E+03	7.5201E+03	7.5528E+03	4.8487E+03	7.8979E+03	7.5924E+03	5.6670E+03	6.7370E+03	**4.3864E+03**	4.4435E+03
	Std	1.6736E+03	6.9889E+02	5.7086E+02	1.4065E+03	5.0758E+02	**3.9663E+02**	1.2201E+03	1.4171E+03	6.8023E+02	5.3056E+02
F11	Ave	2.7282E+03	9.4776E+03	1.5164E+03	1.2204E+03	2.7947E+03	1.2785E+03	2.0257E+03	2.0060E+03	1.2107E+03	**1.2028E+03**
	Std	1.2034E+03	4.2375E+03	3.4455E+02	3.5568E+01	9.7408E+02	**3.3403E+01**	1.7415E+03	1.0392E+03	3.5638E+01	4.9806E+01
F12	Ave	9.1309E+07	5.4014E+08	9.2198E+07	5.5773E+06	1.6924E+09	1.3241E+06	6.4970E+08	6.7126E+07	1.2992E+06	**8.5253E+05**
	Std	8.7190E+07	3.3469E+08	1.1683E+08	3.4569E+06	1.0291E+09	8.5949E+05	2.2918E+09	1.5719E+08	9.7540E+05	**7.3850E+05**
F13	Ave	3.5307E+07	1.0068E+07	5.8931E+06	2.6233E+04	2.8607E+08	**1.9515E+04**	1.3775E+08	1.1744E+07	2.4931E+04	2.1536E+04
	Std	7.3177E+07	7.8142E+06	1.0010E+07	1.1490E+04	2.3420E+08	**9.8885E+03**	6.3955E+08	2.9007E+07	1.9700E+04	1.5896E+04
F14	Ave	6.4912E+05	2.0136E+06	1.1196E+05	1.6227E+04	1.2815E+05	2.0133E+03	4.8014E+04	3.0928E+05	3.8287E+04	**1.9169E+03**
	Std	7.9568E+05	2.3133E+06	1.0316E+05	1.3961E+04	1.9872E+05	**4.9927E+02**	1.7511E+05	4.7635E+05	3.8631E+04	1.7890E+03
F15	Ave	1.3374E+06	8.9547E+06	2.7184E+05	1.3623E+04	1.3103E+06	**4.5081E+03**	6.8261E+04	8.0061E+06	1.5806E+04	4.9744E+03
	Std	2.9560E+06	1.9025E+07	4.7814E+05	2.7325E+03	2.3689E+06	**2.2189E+03**	1.7324E+05	3.8027E+07	2.3608E+04	3.1900E+03
F16	Ave	2.6209E+03	4.3836E+03	2.9657E+03	2.9052E+03	3.8503E+03	3.0711E+03	3.0919E+03	3.2802E+03	2.4044E+03	**2.2420E+03**
	Std	4.0545E+02	7.1324E+02	2.7630E+02	3.1711E+02	3.2531E+02	**1.7303E+02**	4.9562E+02	4.0631E+02	3.6765E+02	3.1093E+02
F17	Ave	2.0962E+03	2.8033E+03	2.1752E+03	2.2647E+03	2.6719E+03	2.0707E+03	2.4803E+03	2.6625E+03	1.9741E+03	**1.9372E+03**
	Std	2.0366E+02	2.2382E+02	2.0385E+02	2.6774E+02	2.6189E+02	**9.9814E+01**	3.2042E+02	2.8522E+02	1.5160E+02	1.2415E+02
F18	Ave	1.3310E+06	1.6471E+07	2.0415E+06	1.1381E+05	2.5284E+06	1.5169E+05	4.2037E+05	3.6803E+06	4.8688E+05	**2.8351E+04**
	Std	1.7429E+06	1.8120E+07	2.4120E+06	6.4697E+04	2.8416E+06	1.4709E+05	1.2951E+06	3.8499E+06	4.0472E+05	**2.2834E+04**
F19	Ave	2.1341E+06	2.1691E+07	4.3388E+05	1.8548E+04	1.2736E+07	7.6674E+03	4.7208E+06	4.6345E+06	1.5969E+04	**4.2314E+03**
	Std	5.5505E+06	2.1116E+07	4.1782E+05	3.2079E+04	1.3101E+07	6.0745E+03	1.5245E+07	7.6618E+06	4.2821E+04	**4.9705E+03**
F20	Ave	2.5098E+03	2.9590E+03	2.5433E+03	2.4653E+03	2.8238E+03	2.4189E+03	2.6182E+03	2.7894E+03	**2.2680E+03**	2.2686E+03
	Std	1.8801E+02	2.2231E+02	1.9765E+02	1.6276E+02	2.3151E+02	**1.2319E+02**	2.0509E+02	2.4652E+02	1.3649E+02	1.4425E+02
F21	Ave	2.4063E+03	2.6418E+03	2.5123E+03	2.4465E+03	2.6056E+03	2.4808E+03	2.5432E+03	2.5612E+03	2.3660E+03	**2.3402E+03**
	Std	2.3393E+01	6.2579E+01	2.9483E+01	5.2424E+01	4.7216E+01	**1.4005E+01**	6.0207E+01	5.0822E+01	1.7981E+01	3.7735E+01
F22	Ave	5.3377E+03	7.9756E+03	5.5005E+03	3.2566E+03	5.6194E+03	2.3103E+03	6.3179E+03	4.5387E+03	2.4520E+03	**2.3006E+03**
	Std	1.6868E+03	2.2023E+03	3.1272E+03	1.7246E+03	1.9689E+03	5.1918E+00	1.9333E+03	2.3756E+03	7.3944E+02	**1.4707E+00**
F23	Ave	2.7922E+03	3.1405E+03	2.9348E+03	2.8033E+03	3.0463E+03	2.8430E+03	3.1170E+03	3.0068E+03	2.7256E+03	**2.7015E+03**
	Std	4.8920E+01	8.8062E+01	7.5595E+01	3.9414E+01	6.1828E+01	1.6531E+01	1.2384E+02	9.8268E+01	2.2269E+01	**1.6235E+01**
F24	Ave	2.9656E+03	3.2805E+03	3.0983E+03	2.9570E+03	3.2068E+03	3.0225E+03	3.2320E+03	3.1791E+03	2.8980E+03	**2.8714E+03**
	Std	8.0574E+01	1.0683E+02	5.6880E+01	3.7202E+01	6.2607E+01	2.3517E+01	9.4203E+01	9.5351E+01	2.8179E+01	**1.7784E+01**
F25	Ave	3.0092E+03	3.2152E+03	2.9912E+03	2.9138E+03	3.4230E+03	2.9094E+03	3.1955E+03	2.9912E+03	2.9063E+03	**2.8972E+03**
	Std	4.3760E+01	8.1018E+01	5.6473E+01	2.4012E+01	1.9828E+02	**1.5248E+01**	4.0219E+02	5.5120E+01	1.7554E+01	1.7670E+01
F26	Ave	5.0761E+03	8.6603E+03	5.1375E+03	5.8790E+03	7.5891E+03	4.5077E+03	8.0147E+03	7.1059E+03	4.1725E+03	**4.0294E+03**
	Std	5.4387E+02	1.0451E+03	1.0323E+03	1.5623E+03	1.2175E+03	1.3906E+03	1.5363E+03	8.0317E+02	7.8265E+02	**5.1965E+02**
F27	Ave	3.2695E+03	3.4938E+03	3.2690E+03	3.2915E+03	3.4444E+03	3.2790E+03	3.4184E+03	3.3384E+03	**3.2190E+03**	3.2773E+03
	Std	2.9076E+01	1.4456E+02	4.3752E+01	3.5492E+01	9.6874E+01	1.2717E+01	2.3009E+02	8.7356E+01	**9.8633E+00**	6.0190E+01
F28	Ave	3.4914E+03	3.9226E+03	3.3610E+03	3.2618E+03	4.2293E+03	3.2882E+03	3.8594E+03	3.4425E+03	3.2498E+03	**3.2272E+03**
	Std	1.8200E+02	2.6964E+02	5.2829E+01	**1.7949E+01**	4.2128E+02	3.0045E+01	8.8127E+02	1.0353E+02	3.4882E+01	2.6378E+01
F29	Ave	3.9548E+03	5.3457E+03	4.0880E+03	4.3527E+03	5.1064E+03	4.0618E+03	4.6728E+03	4.3757E+03	3.6654E+03	**3.5726E+03**
	Std	1.5698E+02	4.6247E+02	1.8989E+02	2.8625E+02	5.5070E+02	1.5060E+02	4.9068E+02	3.1159E+02	1.5081E+02	**1.3315E+02**
F30	Ave	1.2005E+07	7.3508E+07	2.6422E+06	7.1489E+05	8.5683E+07	1.2292E+05	1.3768E+07	2.8592E+06	**4.8924E+04**	2.0272E+05
	Std	1.0051E+07	4.6705E+07	1.7591E+06	7.2019E+05	5.1032E+07	**9.6102E+04**	5.2693E+07	5.3691E+06	9.7389E+04	7.2366E+05
Mean		5.37	9.53	5.87	4.20	8.93	3.83	6.83	6.93	2.30	**1.20**
Friedman		5	10	6	4	9	3	7	8	2	**1**

According to the test results of Table 3, among the 30 functions in CEC2017, MSBOA performs the best on 25 functions, ranks second on 4 functions, and third on 1 function. In comparison, the performance distribution of the other 9 algorithms across the 30 functions is as follows: CPO and SBOA rank first on 2 and 3 functions respectively, while PSO, RUN, CPO, and SBOA rank second on 1, 3, 3, and 19 functions respectively. GWO, RUN, CPO, BKA, and SBOA rank third on 7, 5, 10, 1, and 6 functions respectively. From the perspective of rankings, the proposed MSBOA maintains its position in the top three on all 30 functions, demonstrating the stability of its performance.

It is noteworthy that the recently proposed CPO and SBOA have Friedman values of 3.83 and 2.30 respectively, higher than the classical algorithms GWO, WOA, PSO, and RUN with Friedman values of 5.37, 9.53, 5.87, and 4.20 respectively. This indicates that the overall performance of these recently proposed algorithms is superior to these classical algorithms on this problem. Moreover, the Friedman value of MSBOA is 1.20, surpassing all other comparative algorithms, indicating the effectiveness of the four integrated innovative strategies, which enhance the algorithm’s exploration performance and consequently improve its overall performance.

### Statistical analysis

#### Wilcoxon rank sum test

In this subsection, the Wilcoxon rank-sum test ^66^ is employed to assess whether significant differences exist in the performance of the MECOA algorithm, without relying on assumptions of normality. Compared to the traditional t-test, the Wilcoxon test offers greater flexibility, as it remains applicable to data with non-normal distributions or outliers. The test statistic $W$ for the Wilcoxon rank-sum test is defined by Equation (31).

31 $$\begin{array}{c}W=\sum_{i=1}^{{n}_{1}}R\left({X}_{i}\right)\end{array}$$

where $R\left({X}_{i}\right)$ denotes the rank of ${X}_{i}$ among all observations. The test statistic $U$ is calculated by Equation (32).

32 $$\begin{array}{c}U=W-\frac{{n}_{1}\left({n}_{1}+1\right)}{2}\end{array}$$

For larger sample sizes, $U$ is approximately normally distributed by Equation (33)

33 $$\begin{array}{c}\genfrac{}{}{0pt}{}{{\mu }_{U}=\frac{{n}_{1}{n}_{2}}{2}}{{\sigma }_{U}=\sqrt{\frac{{n}_{1}{n}_{2}\left({n}_{1}+{n}_{2}+1\right)}{12}}}\end{array}$$

and the standardized statistic Z is calculated by Equation (34).

34 $$\begin{array}{c}Z=\frac{U-{\mu }_{U}}{{\sigma }_{U}}\end{array}$$

A significance level of 0.05 was adopted to determine whether the results of each MSBOA run exhibited a statistically significant difference from those of other algorithms. Under the null hypothesis (${H}_{0}$), it is assumed that no significant difference exists between the two algorithms. If the $p$-value is less than 0.05, ${H}_{0}$ is rejected, indicating a significant performance difference; otherwise, it is retained.

To further validate the superior performance of MSBOA compared to other benchmark algorithms, the Wilcoxon rank-sum test was employed to conduct a statistical significance analysis of the algorithms’ performance on the CEC2017 benchmark functions. The test results are presented in Table 4, where the "(+/=/-)" indicator represents the number of benchmark functions (out of 30) in which MSBOA performed better than, equal to, or worse than the comparison algorithms, respectively. The data in Table 4 shows that MSBOA exhibits strong performance competitiveness. Specifically, when compared with WOA, NRBO, BKA, and DBO, MSBOA outperformed all four algorithms across all 30 benchmark functions, with no instances of equal or inferior performance, fully demonstrating its comprehensive advantage in complex function optimization scenarios. In comparisons with GWO, PSO, and RUN, MSBOA performed slightly worse on only one benchmark function but maintained a performance lead on the remaining 29 functions, indicating that while it has minor limitations in a few specific function structures, its overall optimization capability is significantly superior to traditional classical algorithms. Even when compared with the relatively high-performing CPO and the original SBOA, MSBOA still outperformed them on 26 and 24 functions, respectively, while underperforming on only 4 and 6 functions, further confirming the performance enhancement achieved by the proposed multi-strategy improvements to the SBOA algorithm.

**Table 4:** Results for various algorithms on the CEC 2017.

Algorithm	GWO	WOA	PSO	RUN	NRBO	CPO	BKA	DBO	SBOA
(+/=/-)	(29/0/1)	(30/0/0)	(29/0/1)	(29/0/1)	(30/0/0)	(26/0/4)	(30/0/0)	(30/0/0)	(24/0/6)

Based on a significance level of 0.05, all the aforementioned performance differences passed the statistical significance test, indicating that MSBOA’s performance advantages are not due to random factors but are stable improvements resulting from its algorithmic structural enhancements. This lays a reliable theoretical foundation for its subsequent application to UAV path planning problems in complex environments.

#### Friedman mean rank test

In this subsection, the Friedman test ^67^ is used to determine the overall ranking of the MECOA algorithm relative to other methods. As a nonparametric approach, the Friedman test is suitable for comparing median performance differences across three or more matched groups. It is particularly well-suited for repeated measures or block designs, and is often employed as a robust alternative to ANOVA when the assumption of normality is violated. The Friedman test statistic is calculated according to Equation (35).

35 $$\begin{array}{c}Q=\frac{12}{nk\left(k+1\right)}\sum_{j=1}^{k}{R}_{j}^{2}-3n\left(k+1\right)\end{array}$$

where $n$ is the number of blocks, $k$ is the number of groups, and ${R}_{j}$ is the rank sum for $j$-th group. When $n$ and $k$ are large, $Q$ follows approximately a ${\chi }^{2}$ distribution with $k-1$ degrees of freedom.

According to the Friedman mean rank test results in Table 5, MSBOA achieved an average rank (M.R) of 1.20 on the CEC2017 benchmark functions, with a total rank (T.R) of first place, demonstrating significant superiority over other comparison algorithms. Traditional algorithms such as GWO (M.R=5.37, T.R=5) and WOA (M.R=9.53, T.R=10), as well as recent advanced algorithms like CPO (M.R=3.83, T.R=3) and the original SBOA (M.R=2.30, T.R=2), all exhibited lower average and total rankings compared to MSBOA. These results fully confirm that MSBOA delivers superior comprehensive performance when handling unimodal, multimodal, hybrid, and composition optimization problems covered by the CEC2017 benchmark suite. The multi-strategy improvements effectively enhance the algorithm’s overall optimization capability, providing reliable support for its subsequent application to UAV path planning in complex environments.

**Table 5:** Friedman mean rank test result.

Algorithm	GWO	WOA	PSO	RUN	NRBO	CPO	BKA	DBO	SBOA	MSBOA
M. R	5.37	9.53	5.87	4.20	8.93	3.83	6.83	6.93	2.30	**1.20**
T. R	5	10	6	4	9	3	7	8	2	**1**

### Experiment in UAV path planning

#### Scenario Settings

The scenarios used for evaluation are based on real Digital Elevation Model (DEM) (terrain resolution:$1045\times 879$)maps derived from LiDAR sensors ^68^. Initially, two terrains from Christmas Island, Australia, representing both land and ocean topographies, are selected. When the altitude is below sea level, it is uniformly set to 0. Subsequently, different regions with varying complexities are enhanced to generate eight benchmark scenarios. In these eight scenarios, the number and positions of threatening objects (represented by red cylinders) vary, implying different levels of complexity in solving, thus effectively assessing the algorithm’s ability to find the optimal path.

In this study, the number of waypoints is set to $n = 12$, corresponding to 10 line segments^55^. In each comparison, the parameter settings for all algorithms are as shown in Table 2, and each algorithm is run 50 times to obtain the mean and standard deviation. Additionally, we employ the non-parametric Wilcoxon rank-sum (*P*-value) test to compare differences between algorithms ^69^. "$N/A$" indicates that the results of two algorithms are too similar to make a significant judgment. If the result exceeds 0.05, we consider there is no significant difference between the algorithms and highlight accordingly. Conversely, significant differences exist between the two algorithms.

#### Compared with the classical algorithm

**Fig. 7:**
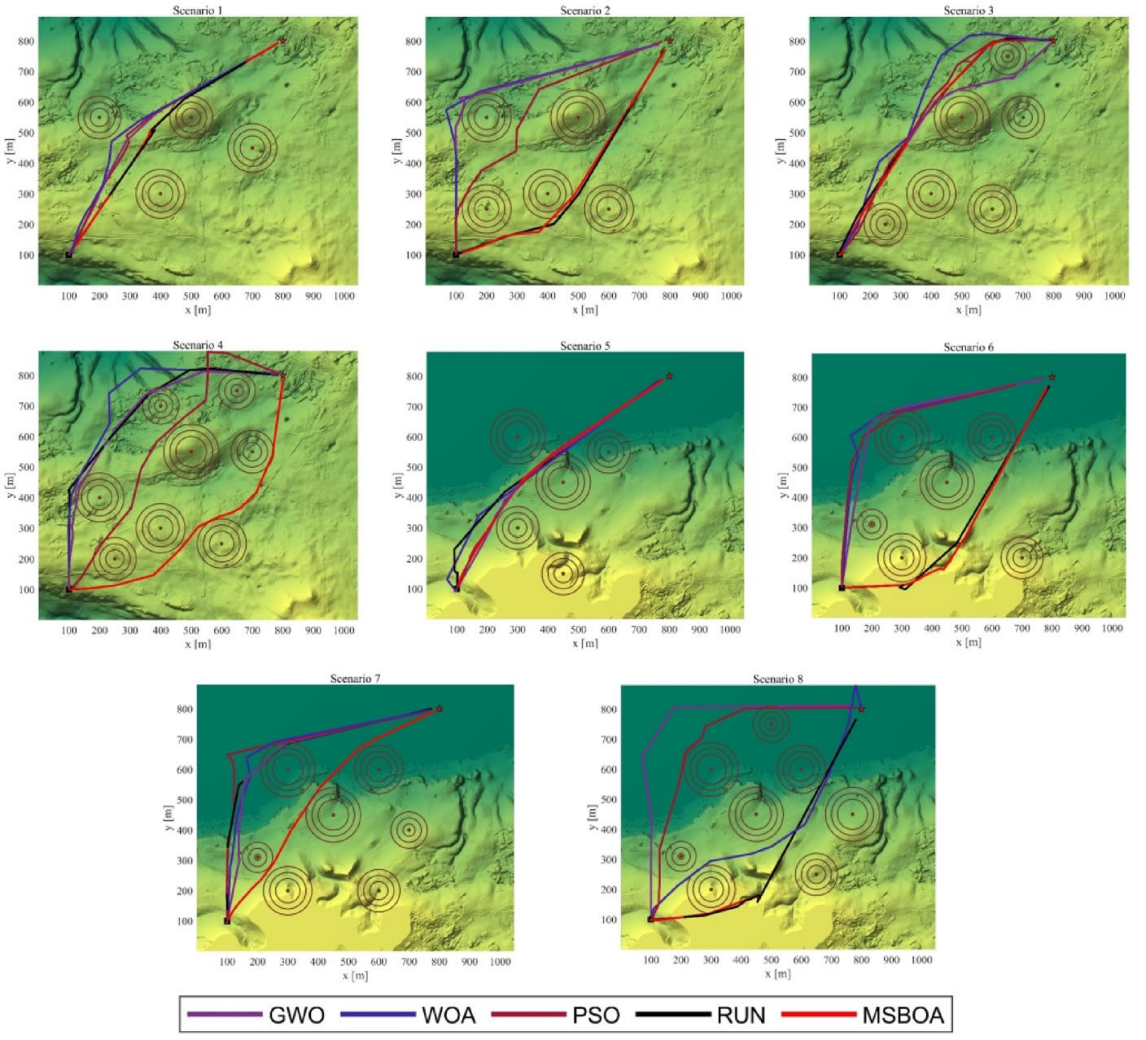
Top view of the paths generated by the classical algorithms.

The top-down view of the paths generated by MSBOA and classical algorithms is depicted in Fig. 7. It can be observed that all algorithms are capable of generating feasible paths that satisfy requirements for path length, threats, turning angles, climb/descent angles, and altitude. However, their optimality varies depending on the scenario. For simple scenarios 1, 2, 5, and 6, all algorithms exhibit good convergence, albeit with slightly different fitness values. The test values in Table 6 also indicate that GWO is not statistically significant in scenarios 1, 2, 5, and 6. However, for complex scenarios 3, 4, 7, and 8, their performances differ significantly. MSBOA is able to achieve near-optimal solutions, while GWO, PSO, and SBOA converge to relatively good solutions only. WOA fails to find high-quality solutions. Table 6 displays the mean, standard deviation, and non-parametric Wilcoxon rank-sum test values, further confirming the fitness values of the tests. It suggests that MSBOA statistically achieves the best fitness across most tested scenarios, while GWO and PSO are effective only in simple scenarios. RUN introduces relatively good results across all scenarios, reflecting stable convergence with minor deviations.

**Table 6:** Experimental results of MSBOA and classical algorithms.

Scenario	Algorithms	GWO	WOA	PSO	RUN	MSBOA
Scenario-1	Ave	5.1887E+03	5.9470E+03	5.3046E+03	5.2715E+03	**5.1832E+03**
	Std	2.3677E+01	2.8424E+02	4.0908E+01	1.2963E+02	**1.6239E+01**
	*P*-value	**2.9820E-01**	3.2827E-21	9.0429E-22	4.7680E-04	N/A
Scenario-2	Ave	5.1889E+03	6.0024E+03	5.3279E+03	5.2841E+03	**5.1778E+03**
	Std	3.5850E+01	2.5536E+02	6.9074E+01	1.0174E+02	**4.9637E+00**
	*P*-value	**9.8280E-02**	4.9399E-25	3.6911E-17	3.9757E-07	N/A
Scenario-3	Ave	5.4028E+03	7.3787E+03	5.8807E+03	5.5287E+03	**5.3392E+03**
	Std	**9.2381E+00**	7.3500E+02	1.4599E+02	1.1390E+02	9.1806E+01
	*P*-value	3.7557E-04	9.8728E-22	1.9065E-24	2.0206E-09	N/A
Scenario-4	Ave	7.6511E+03	9.1480E+03	7.5494E+03	6.2630E+03	**5.6407E+03**
	Std	2.0746E+03	1.2242E+03	1.1632E+03	3.5793E+02	**2.3469E+02**
	*P*-value	2.0633E-06	3.5965E-22	2.7321E-12	7.0326E-11	N/A
Scenario-5	Ave	5.1699E+03	5.6224E+03	5.2810E+03	5.2398E+03	**5.1646E+03**
	Std	1.8266E+01	1.7672E+02	7.8420E+01	6.1714E+01	**1.2234E+01**
	*P*-value	**1.9676E-01**	1.8118E-20	5.3810E-11	1.7036E-08	N/A
Scenario-6	Ave	5.2487E+03	7.1671E+03	5.5199E+03	5.3674E+03	**5.2431E+03**
	Std	1.5492E+01	5.6053E+02	6.9952E+01	1.5417E+02	**1.1192E+01**
	*P*-value	**1.1392E-01**	2.3916E-26	3.3132E-29	4.6354E-05	N/A
Scenario-7	Ave	6.3039E+03	7.5116E+03	6.4274E+03	5.7638E+03	**5.6485E+03**
	Std	5.5734E+02	8.9572E+02	2.3563E+02	1.9580E+02	**1.0174E+02**
	*P*-value	3.7836E-08	2.5735E-16	9.9560E-24	5.8707E-03	N/A
Scenario-8	Ave	7.6044E+03	8.3451E+03	7.3637E+03	5.7163E+03	**5.6651E+03**
	Std	1.6759E+03	1.2868E+03	1.0678E+03	1.9396E+02	**2.7398E+01**
	*P*-value	3.7647E-08	1.8999E-16	4.0088E-12	**1.5724E-01**	N/A

**Fig. 8:**
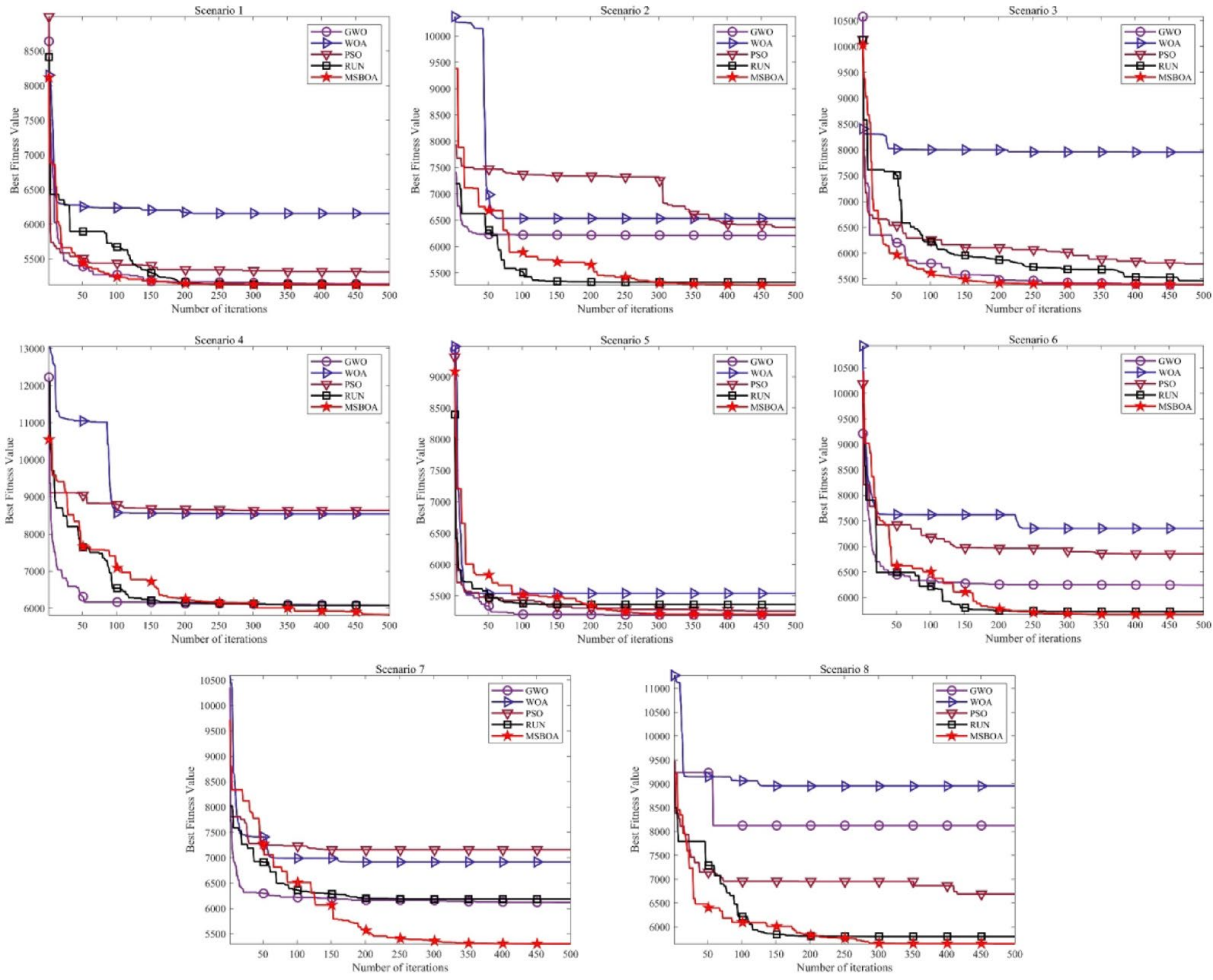
Best fitness values over iterations of the classical algorithms.

Figure 8 provides a closer observation of the behavior of these classical algorithms by displaying their best fitness values during iterations. From the graph, it can be observed that in simple scenarios 1 and 5, GWO obtains good fitness values with fast convergence speed. However, for other complex scenarios, it is prone to getting trapped in local optima, failing to achieve high-quality solutions, similar to other algorithms. On the other hand, MSBOA consistently obtains high-quality solutions across all eight scenarios. Although in scenarios 2, 4, and 6, its convergence speed in the early iterations is slower, it does not get trapped in local optima like SBOA, GWO, and WOA, but continues to search for higher-quality solutions. This demonstrates the effectiveness of the four embedded improvement strategies, enhancing the overall performance of MSBOA, capable of obtaining relatively smooth and shorter paths in complex UAV path planning scenarios.

#### Compare with recent algorithms

To further evaluate the performance of MSBOA, we compare its efficacy with other recently proposed advanced metaheuristic algorithms, namely Newton-Raphson-based optimizer (NRBO), Crested Porcupine Optimizer (CPO), Black-winged Kite Algorithm (BKA), Dung Beetle Optimizer (DBO), and Secretary Bird Optimization Algorithm (SBOA). The parameter settings for each algorithm adhere to the standards specified in their respective original literature, as illustrated in Table 2 above.

Table 7 presents the fitness results. It is evident that the performance of MSBOA surpasses that of other algorithms. Specifically, across all scenarios, except for the standard deviation in scenarios 1 and 3 where SBOA slightly outperforms, both the mean and standard deviation of MSBOA excel those of the comparative algorithms. Particularly noteworthy is its significantly superior mean performance in complex scenarios 3, 4, 5, and 8 compared to other algorithms.

**Table 7:** Experimental results of MSBOA and recent algorithms.

Scenario	Algorithms	NRBO	CPO	BKA	DBO	SBOA	MSBOA
Scenario-1	Ave	5.3058E+03	5.3536E+03	5.2674E+03	5.3888E+03	5.1886E+03	**5.1832E+03**
	Std	5.0491E+01	9.8437E+01	1.4735E+02	2.7379E+02	**6.7417E+00**	1.6239E+01
	*P*-value	2.4127E-18	3.4279E-13	2.8893E-03	1.2707E-04	**9.8516E-02**	N/A
Scenario-2	Ave	5.3141E+03	5.3526E+03	5.2595E+03	5.3667E+03	5.1889E+03	**5.1778E+03**
	Std	4.3190E+01	1.1848E+02	1.1325E+02	1.9946E+02	7.9227E+00	**4.9637E+00**
	*P*-value	2.1087E-24	4.6498E-11	2.1733E-04	2.8638E-06	2.1319E-08	N/A
Scenario-3	Ave	5.7763E+03	6.0690E+03	5.5820E+03	5.8913E+03	5.3825E+03	**5.3392E+03**
	Std	1.3744E+02	2.4207E+02	1.5265E+02	4.2291E+02	**7.1347E+01**	9.1806E+01
	*P*-value	6.2980E-21	3.2995E-22	4.8326E-10	3.0738E-09	4.5875E-02	N/A
Scenario-4	Ave	6.3840E+03	6.6591E+03	6.6031E+03	6.6960E+03	5.7050E+03	**5.6407E+03**
	Std	7.3160E+02	4.4631E+02	1.0714E+03	6.6061E+02	2.7933E+02	**2.3469E+02**
	*P*-value	1.8818E-06	6.4600E-16	1.1287E-05	2.3799E-11	**3.3844E-01**	N/A
Scenario-5	Ave	5.2649E+03	5.2974E+03	5.2364E+03	5.3124E+03	5.1863E+03	**5.1646E+03**
	Std	5.1451E+01	1.0167E+02	8.1482E+01	1.3726E+02	1.3016E+01	**1.2234E+01**
	*P*-value	7.4126E-15	1.9621E-09	1.2806E-05	2.2026E-07	1.1619E-08	N/A
Scenario-6	Ave	5.5700E+03	5.6792E+03	5.3552E+03	5.4919E+03	5.2645E+03	**5.2431E+03**
	Std	1.4700E+02	3.2363E+02	1.3426E+02	3.1431E+02	1.6752E+02	**1.1192E+01**
	*P*-value	1.4438E-17	6.8583E-10	2.7550E-05	5.9478E-05	**4.8954E-01**	N/A
Scenario-7	Ave	5.8245E+03	6.2561E+03	5.7730E+03	5.9283E+03	5.7894E+03	**5.6485E+03**
	Std	2.9183E+02	4.1072E+02	4.8131E+02	4.5431E+02	2.9906E+02	**1.0174E+02**
	*P*-value	2.8253E-03	1.0328E-10	**1.7117E-01**	1.6995E-03	1.7649E-02	N/A
Scenario-8	Ave	5.9652E+03	6.4700E+03	5.9811E+03	5.9984E+03	5.7967E+03	**5.6651E+03**
	Std	4.3999E+02	4.9901E+02	7.3521E+02	4.8964E+02	3.8479E+02	**2.7398E+01**
	*P*-value	4.3953E-04	2.6176E-12	2.2067E-02	4.4801E-04	**6.6698E-02**	N/A

**Fig. 9:**
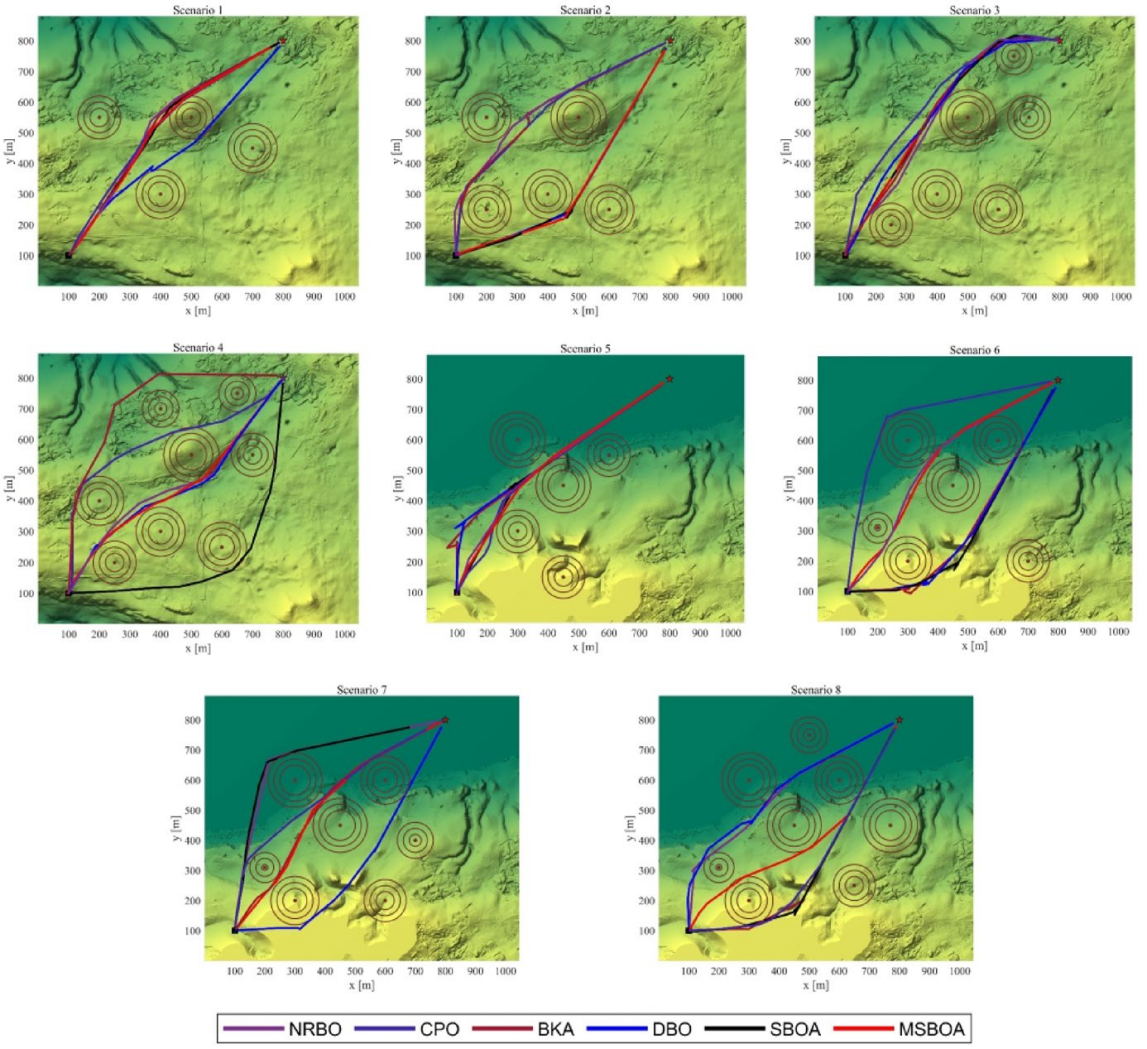
Top view of the paths generated by the recent algorithms.

Figure 9 provides a top-down view of the generated paths. It is evident that all algorithms are capable of generating collision-free paths. However, MSBOA consistently produces the smoothest and shortest paths in most cases. In contrast, the original SBOA only achieves relatively smooth and short paths in simple environments; its performance significantly deteriorates in complex environments, failing to generate the smoothest and shortest paths.

**Fig. 10:**
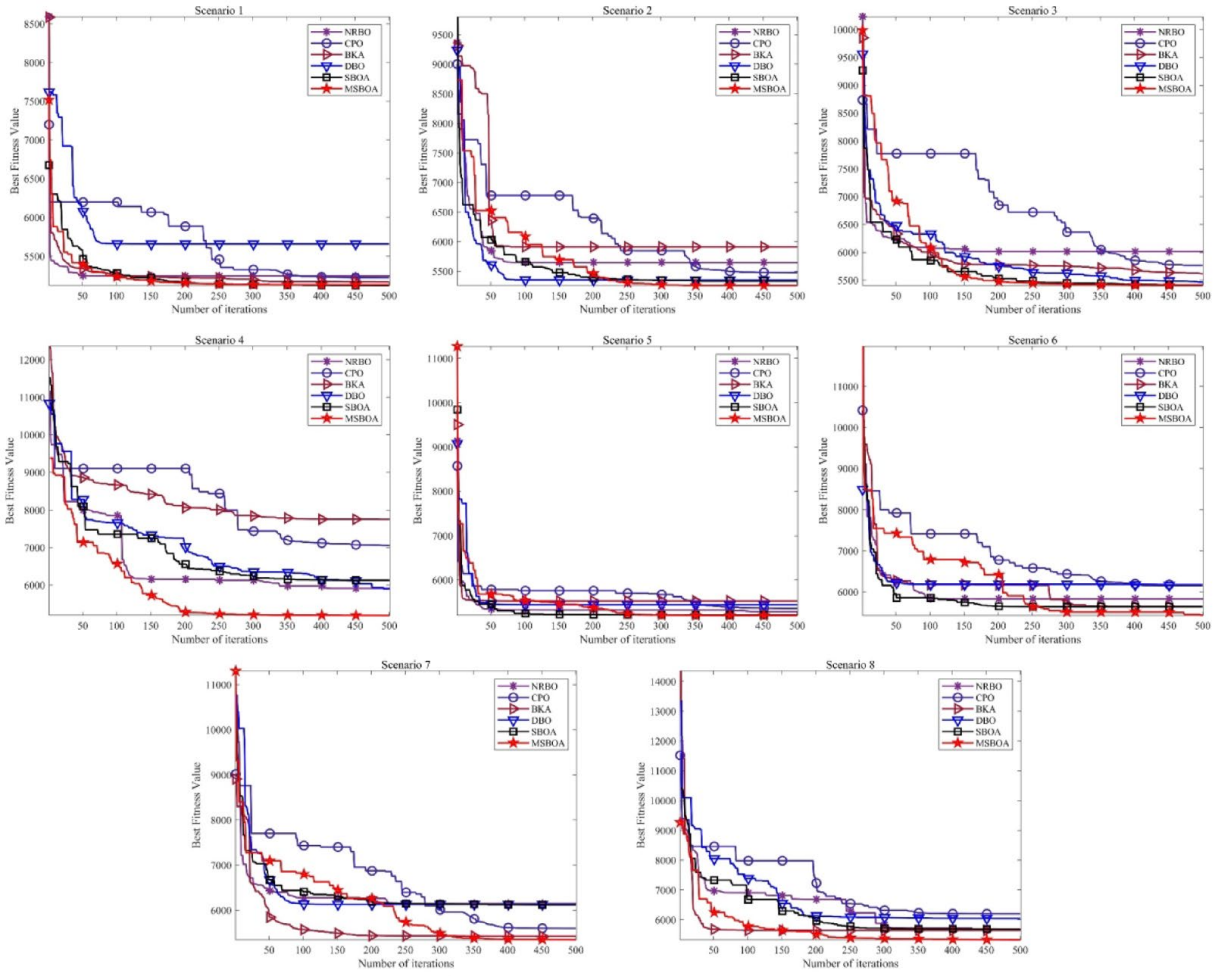
Best fitness values over iterations of the recent algorithms.

Figure 10 illustrates the variation of the best fitness value during iterations. It is apparent that DBO quickly converges to premature solutions as the number of iterations increases. Due to its limited exploration capability, CPO exhibits slow convergence and is prone to getting trapped in local optima during later iterations. SBOA demonstrates stable convergence but also falls into local optima towards the end of iterations, failing to attain higher-quality solutions. In contrast, MSBOA exhibits sufficiently rapid convergence, attributed to the four effective improvement strategies introduced, thereby enhancing its convergence performance.

## Summary and prospect

This paper proposes an improved Secretary Bird Optimization Algorithm – the Multi-Strategy Secretary Bird Optimization Algorithm (MSBOA) – for solving unmanned aerial vehicle (UAV) path planning problems, with a focus on ensuring path safety and feasibility. The design of the cost function incorporates constraints related to optimality, safety, and feasibility simultaneously. MSBOA is devised by integrating four innovative strategies: the Pooling mechanism, Dynamic Fitness Distance Balance Strategy, Centroid Opposition-based Learning with Greedy Selection, and Preferential Selecting Search Strategy.

To validate the effectiveness of MSBOA, comparisons are conducted with nine other algorithms on the CEC2017 benchmark functions. Results indicate that MSBOA exhibits superior convergence performance. Comparative analyses on eight benchmark scenarios generated from DEM maps demonstrate that MSBOA consistently achieves optimal-quality paths in most cases, along with high robustness. Comparisons with other metaheuristic algorithms also confirm the outstanding performance of MSBOA.

As the No Free Lunch theorem posits, no metaheuristic algorithm can outperform all others across all problem domains, and MSBOA is no exception, with its limitations particularly evident in handling certain CEC2017 functions. Its inferior performance compared to SBOA and CPO on F10, F27, and F30 stems from mismatches between its core mechanisms and the specific characteristics of these functions, which also points to directions for targeted improvements:

For F10, which features a rugged landscape with the global optimum located at the search space boundary, MSBOA’s underperformance is primarily attributed to its centroid reverse learning strategy. This strategy tends to generate reverse solutions concentrated in the central region of the search space, resulting in insufficient exploration of boundary areas. In contrast, SBOA’s Levy flight (which enables occasional long jumps to boundary regions) and CPO’s adaptive local search (which intensifies exploitation near promising boundary areas) are better suited to this function’s trait. To address this, future iterations could modify the centroid reverse learning mechanism—for instance, by adjusting the centroid calculation to incorporate boundary information or introducing a probability weight that biases reverse solution generation toward boundary regions, thereby enhancing boundary exploration.

For composite functions F27 and F30, which consist of fragmented sub-regions with distinct optima, MSBOA’s pooling mechanism leads to computational redundancy when adapting to these dispersed sub-regions. This is because the pooling strategy aggregates information across the entire population, diluting the specificity required to respond to fragmented landscapes. In comparison, SBOA and CPO’s simpler update rules (which prioritize local information exchange) allow faster adaptation to sub-region characteristics. Potential improvements here could involve refining the pooling mechanism—such as dynamically partitioning the population into sub-groups corresponding to detected sub-regions, or introducing adaptive weights to the pooling process that emphasize local sub-region information, thus reducing redundancy and accelerating adaptation to composite function structures.

In some UAV path planning scenarios, MSBOA lags behind GWO, BKA, and SBOA in early convergence speed. This is because it employs centroid reverse learning to avoid premature convergence—a critical design choice for complex environments with dense threats—but this comes at the cost of slower early exploration. In contrast, GWO, BKA, and SBOA rely on fast local exploitation or stage-wise hunting strategies, which enable rapid early convergence in simple, sparse-threat scenarios. To balance this, future work could explore a dynamic strategy adjustment mechanism: adopting more aggressive local exploitation in the early stages for sparse-threat environments while retaining the current anti-premature convergence mechanisms for complex environments, thus improving adaptability across scenario types.

To further expand the theoretical depth and practical application value of the Multi-Strategy Secretary Bird Optimization Algorithm (MSBOA), subsequent research will be conducted across multiple dimensions. On one hand, for multi-UAV cooperative operation scenarios, MSBOA will be integrated with a distributed optimization framework to design a multi-objective cost function that incorporates task completion time, fuel consumption, and cooperative collision avoidance distance. Simultaneously, a communication constraint model will be introduced to adapt to the data transmission characteristics between UAVs, enabling efficient multi-UAV cooperative path planning. On the other hand, focusing on the demands of dynamic online environments, a dynamic threat prediction model based on Kalman filtering or LSTM neural networks will be constructed. An incremental population update strategy will be designed to reduce computational redundancy during environmental changes, ensuring that UAVs can quickly adjust their paths in scenarios involving moving threats and sudden obstacles. Additionally, a Hardware-in-the-Loop (HIL) test platform integrating UAV flight control systems, simulation software, and environmental sensors will be developed. This platform will validate the path tracking accuracy and robustness of MSBOA in an environment simulating real-flight physical characteristics. Through outdoor physical experiments using small consumer-grade UAVs, the algorithm will be advanced from software simulation to practical application. Furthermore, the step-size control mechanism of the priority selection search strategy will be optimized to enhance adaptability to high-dimensional composite problems. The algorithm will also be extended to cross-domain scenarios such as unmanned vehicle path planning and photovoltaic system optimization. Relevant source code and experimental toolkits will be open-sourced to promote academic exchange and industry application.

## Data Availability

All data generated or analysed during this study are included in this published article.
